# Toxicity of immune checkpoint inhibitors and tyrosine kinase inhibitor combinations in solid tumours: a systematic review and meta-analysis

**DOI:** 10.3389/fonc.2024.1380453

**Published:** 2024-07-15

**Authors:** David O’Reilly, Caroline L. O’Leary, Aislinn Reilly, Min Yuen Teo, Grainne O’Kane, Lizza Hendriks, Kathleen Bennett, Jarushka Naidoo

**Affiliations:** ^1^ Medical Oncology, Beaumont Hospital, Dublin, Ireland; ^2^ Department of Medicine, School of Health Sciences, RCSI University of Medicine and Health Sciences, Dublin, Ireland; ^3^ Medical Oncology, Bon Secours Hospital, Dublin, Ireland; ^4^ Department of Medicine, Memorial Sloan Kettering Cancer Centre, New York, NY, United States; ^5^ HOPE Directorate, Trinity St. James’s Cancer Institute, Trinity College Dublin, Dublin, Ireland; ^6^ Department of Pulmonary Diseases, GROW-School for Oncology and Developmental Biology, Maastricht University Medical Center, Maastricht, Netherlands; ^7^ Data Science Centre, School of Population Health, RCSI University of Medicine and Health Sciences, Dublin, Ireland; ^8^ Thoracic Oncology, Sidney Kimmel Comprehensive Cancer Centre at Johns Hopkins University, Baltimore, MD, United States

**Keywords:** tyrosine kinase inhibitors, immunotherapy, toxicity, immune related adverse events, immune checkpoint blockade

## Abstract

The combination of immune checkpoint inhibitors (ICIs) and tyrosine kinase inhibitors (TKIs) can be associated with significant toxicity. We performed a systematic review and meta-analysis of the toxicity of combination treatment of ICIs with TKIs (ICI + TKI) in clinical trials with solid organ malignancies. Our primary endpoint explored the incidence of grade 3 - 5 (G3-5) treatment-related toxicity and our secondary endpoints included the incidence of toxicity by treatment type, disease type and studies with run-in strategies. A total of 9750 abstracts were identified, of which 72 eligible studies were included. The most common disease types were non-small cell lung cancer (n=8, 11.1%), renal cell carcinoma (n=10, 13.8%) and hepatobiliary cancers (n=10, 13.8%). The overall incidence of G3-5 toxicity was 56% (95% CI = 50% – 61%). The most common TKIs combined with ICIs in this analysis were multi-targeted TKIs (n = 52, 72%), VEGF specific (n = 9, 12.5%), or oncogene-targeting TKIs (*EGFR, ALK, BRAF, MEK*) (n =11, 15.3%). Oncogene-targeted TKIs were associated a higher incidence of rashes and immune related adverse events (irAEs) and lower incidence of hypertension. In studies which used a TKI ‘run-in’ to mitigate toxicity, the pooled estimate of G3-5 toxicity was 71% (95% CI 57-81%). Almost half of studies (48%) omitted the incidence of G3-5 irAEs. Our work suggests that the majority of patients who receive ICI-TKI combinations will experience high grade toxicity (G3-G5) and that toxicity may be specific to TKI partner (Oncogene targeted TKIs: Rash, irAEs; VEGF/Multitargeted: Hypertension). These data did not suggest that a TKI ‘run-in’ was associated with a lower incidence of G3-5 toxicity. Reporting of irAEs was inconsistent supporting the need for harmonisation of adverse event reporting to include onset, duration and treatment.

**Systematic review registration:**

https://www.crd.york.ac.uk/prospero/, identifier CRD42022367416.

## Introduction

Immune checkpoint inhibitors (ICIs) have resulted in improved outcomes for patients with solid organ tumours. However, long-term survival ranges from over 50% amongst patients with advanced melanoma treated with nivolumab plus ipilimumab, to 10 – 30% among those with advanced non-small cell lung cancer (NSCLC) (1–4). Consequently a focus of research is to incorporate novel targeted therapies in combination with ICIs in order to improve response rates and patient outcomes.

Tyrosine kinase inhibitors (TKIs) incorporate a broad range of small molecule therapeutics which may target oncogenes (e.g. Epidermal Growth Factor Receptor, *EGFR*) or other targets in the tumour microenvironment (e.g. Vascular Endothelial Growth Factor, *VEGF*). Oncogenic driver alterations are often associated with suppressive immune microenvironments (5). It is therefore postulated that TKIs may induce anti-tumour immune responses by increasing tumour immunogenicity (5). For these reasons, combination strategies with ICIs and TKIs to maximize therapeutic efficacy, have been investigated in a number of diseases including melanoma, renal cell carcinoma (RCC) and NSCLC (6–8). Additional to approaches involving TKI/ICI combinations include biomarker discovery to identify ICI efficacy and novel immunotherapy approach which may augment ICI efficacy (7, 8).

However, the goal of therapeutic synergy can be complicated by treatment related toxicity. Reports have previously outlined notable toxicities include severe hepatotoxicity with sequential ICI and KRAS_G12C_ inhibitors (sotorasib) and endocrinopathies when ICIs are combined with lenvatinib (9, 10). Additionally, it has been shown that the combination of Lenvatinib and pembrolizumab is associated with a high incidence of fatigue/diarrhoea (11). In clinical practice, it can be difficult to differentiate between a non-immune related adverse event and an immune related AE when patients are receiving ICI/TKI combinations. Given that TKIs can inactivate tumour-associated immunosuppression, this may be the mechanism by which there is an increase in immune-related adverse events (irAEs). However, strategies aimed at minimising toxicity remain ill-defined. Run-in periods with TKIs prior to ICIs or using lower doses of TKIs have been investigated in prospective studies, with a limited biologic basis for this approach (9, 12).

Taken together, there is limited prospective data available to determine the optimal strategy of combining ICIs with TKIs. Given the paucity of data, we seek to assess the safety of TKI/ICI combinations by assessing the spectrum of toxicities when ICIs are combined with TKI’s across tumour types, the toxicities that occur by tumour types and regimen, and the evidence to date involving run-in strategies. These data would then contribute to the optimum combining of ICIs with TKIs, based on toxicity considerations.

## Methodology

### Guidelines

In this review, the Preferred Reporting items for Systematic Reviews and Meta-analyses (PRISMA) guidelines were used and a study protocol (PROSPERO, CRD42022367416) uploaded to an international registry (13).

### Endpoints

The primary endpoint of the study was the incidence of grade 3 - 5 toxicities (G3-5 toxicity) by Common Terminology Criteria for Adverse Events (14). Secondary objectives include identifying factors that associate with high incidences of toxicity including disease type, choice of combination, and utilizing novel approaches including run-in strategies to mitigate toxicity.

### Eligibility criteria

Clinical trials which included anti-cancer treatment with a TKI and ICI for a solid tumour malignancy, were eligible for inclusion. Patients in the included studies may have received >1 TKI or >1 ICI (e.g. vemurafenib plus cobimetinib). Studies in which patients were treated with TKI + ICI + other agent (e.g. cytotoxic therapy) were excluded. Lastly, phase I studies involving dose-escalation cohorts were also excluded.

### Search strategy and study selection

The search strategy utilised the following search terms; Immune checkpoint inhibitors OR immune checkpoint inhibitor, Tyrosine kinase inhibitor OR protein-tyrosine kinases and Neoplasms OR carcinoma OR cancer. In addition to these terms, we also used the MESH terms; Humanized/adverse effects Antineoplastic Agents/therapeutic use Antineoplastic Combined Chemotherapy Protocols/adverse effects. Citations from published work were imported and de-duplicated using Endnote. Forward and backward citation chasing was completed to minimize the possibility of missing relevant studies. MEDLINE, EMBASE, Cochrane Database of Systematic Review and Central Registry of Clinical Trials were searched for publications from 16/8/2002 to 16/8/2022. Conference proceedings (abstracts) were considered eligible and included in our search.

Titles and abstract screening were performed independently by two review authors (COL; AR) to identify potentially eligible studies. Full-text manuscripts identified as potentially eligible were retrieved and independently assessed for eligibility by two review authors (COR, AR). Any disagreement between reviewers over the eligibility of studies were resolved by consensus after discussion with a third reviewer (DOR).

### Data collection and quality assessment

Extracted information included study setting; study population and participant demographics, study methodology; sample size; inclusion and exclusion criteria; details of the intervention and control conditions; recruitment and study completion rates; incidence of symptom toxicity (e.g. diarrhoea, shortness of breath (SOB), rash, liver enzyme abnormalities, fatigue) and irAEs in both intervention and control arms.

To facilitate the assessment of possible risk of bias for each study, we collected information regarding bias of included studies using the Crowe Critical Appraisal Tool (CAT) (15). Domains included in the CAT tool include Introduction, Design, Sampling, Data Collection, Ethical Matters, Results and Discussion.

### Data collection and synthesis methods

We performed descriptive statistics on our study results. Given that some studies did not report all adverse events, the denominator reflects the total number of patients in which the specific toxicity was reported and is different for different toxicities.

The study results were synthesised using a random-effects meta-analysis, with standardised incidence rate ratios for binary outcomes. In reference to our choice of a random-effects meta-analysis, we chose this model (as opposed to a fixed effect model) given that we anticipated that there would be heterogeneity in the included studies. We calculated 95% confidence intervals (95% CI) and two-sided p-values. With sufficient studies available for pooling (minimum of five), we performed meta-analysis by tumour type/organ system (NSCLC, RCC, Hepatobiliary), and treatment regimen (Lenvatinib plus Pembrolizumab, Nivolumab plus Cabozantinib, Axitinib plus Avelumab, ICI + Oncogene-targeted TKI). Hepatobiliary (HPB) tumours include patients with primary hepatocellular carcinomas (HCC) or biliary tract cancers (BTCs). An oncogene-targeted TKI referred to TKIs directed at a specific oncogenic alteration known to be aberrant in the tumour type of interest [e.g. *EGFR, Anaplastic Lymphoma Kinase (ALK*)]. Heterogeneity was assessed using both the Cochran’s Q (chi-squared, χ^2^ statistic), H-squared (H^2^) and the I-squared (I^2^) statistic. H^2^ describes the relative excess in Q over its degrees of freedom as a measure of the extent of heterogeneity and H^2^= 1 indicates homogeneity of effect. We consider an I-squared value greater than 75% indicative of considerable heterogeneity (16). In a protocol-specified (pre-planned) analysis, we investigated the overall incidence of grade 3-5 (G3-5 as per CTCAE) toxicity with the use of concurrent versus TKIs with ICI run-in (14). We conducted sensitivity analyses based on study quality by excluding the poorer quality studies and repeating the analysis for our primary outcome (incidence of G3-5 toxicity in included studies). Finally, to describe heterogeneity across studies, a meta-regression analysis was conducted utilising the covariates of disease group, ICI target, and TKI target (See **Supplementary Appendix** for details on covariates included).

## Results

### Search results

**Figure 1:**
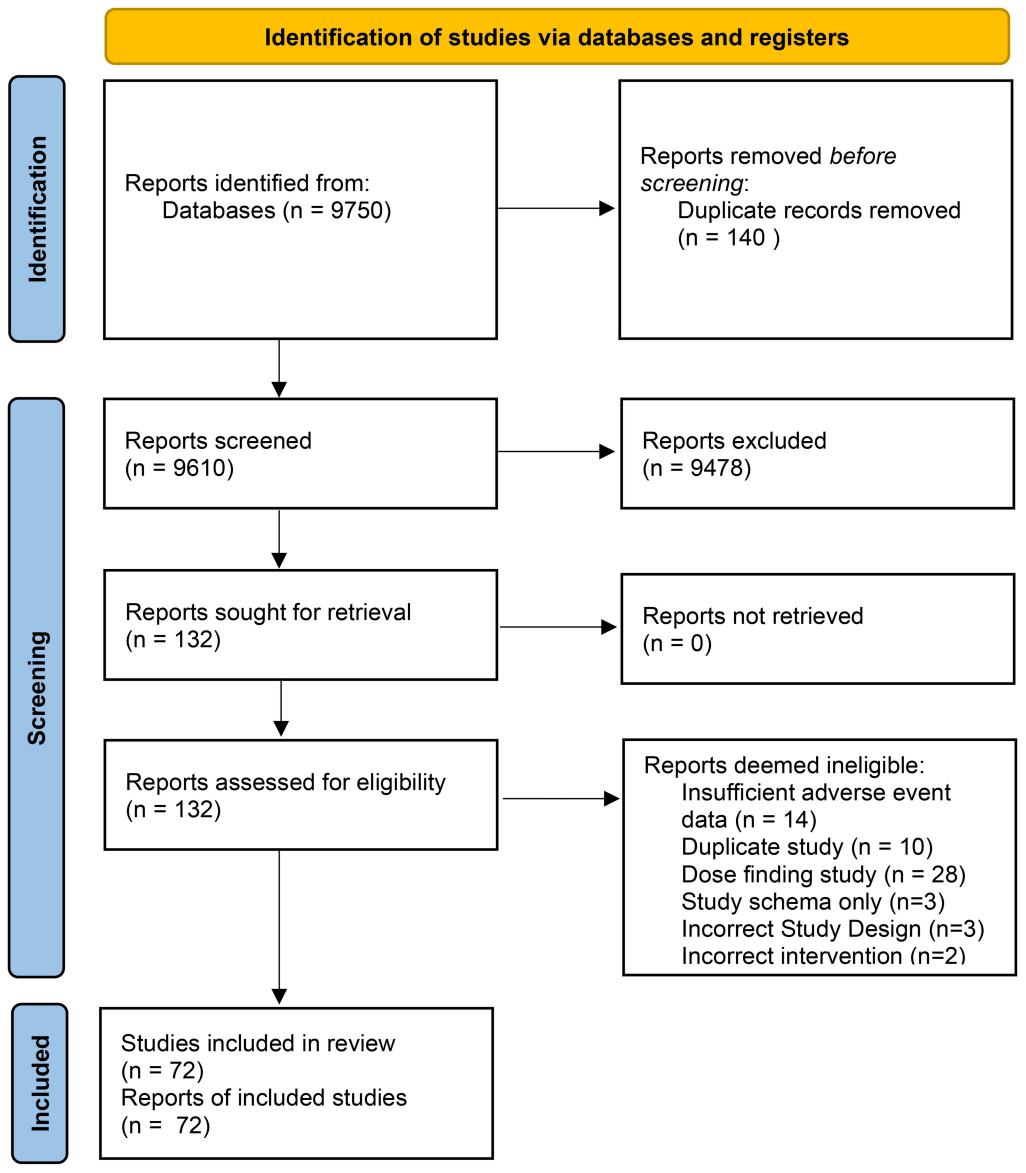
Systematic Review Search results and Eligibility assessment. PRISMA (Preferred Reporting Items for Systematic Reviews and Meta – Analyses) Flow Diagram describing the search results for abstracts & full – texts followed by eligibility assessment.

Our initial search yielded a total of 3348 titles for consideration of inclusion. With the addition of forward and backward citation chasing, a total of 9750 abstracts were identified for potential inclusion (See **Figure 1**: PRISMA flow chart). A total of 132 records were deemed appropriate for full-text review. Upon full-text review a significant number of records were excluded which resulted in a total of 72 eligible studies (See **Supplementary Table 1** for included studies). Studies were excluded for the following reasons; insufficient adverse event reporting (n=14), dose-finding studies (n=28), duplicate studies (n=10) and other reasons (n= 8) (See **Figure 1** for complete breakdown).

### Study and patient characteristics

A total of 9404 patients were treated in 72 studies, of which 5860 (62.3%) received an ICI in combination with a TKI. The remainder of patients (37.4%, 3544/9404) in included studies were not included in our analysis since patients did not receive ICI/TKI (e.g. control arms with single agent TKI). In the identified 72 studies (See **Supplementary Appendix – Table 1**) a total of 20 (27.7%) studies represented abstracts from conference proceedings (See **Table 1**). The median score for the CAT (See **Supplementary Appendix – Table 2**) was 33 (Standard Deviation [SD]=9). For patients receiving ICIs, all studies involved drugs targeting either Programmed Cell Death Protein-1 (PD-1) or Programmed Cell Death Ligand-1 (PD-L1). In 65.2% of studies, patients were treated with a PD-1 inhibitor (47/72) and the remainder were treated with a PD-L1 inhibitor (25/72, 34.7%). In one study, patients received a Cytotoxic T-Lymphocyte Associated Protein 4 (CTLA-4) inhibitor in addition to a PD-1 inhibitor and TKI. The median number of participants in each study was 42 (Range: 10 – 1417). The majority of studies included patients receiving a multi-targeted TKI (52/72, 72.2%) or VEGF-specific TKI (9/72, 12.5%), with the remaining studies including patients receiving TKIs targeting an oncogenic driver alteration, either *BRAF (B-raf), MEK (Mitogen Activated Protein Kinase), EGFR* or *ALK* (11/72, 15.2%). The most commonly used regimens (by number of clinical trials and treated patients) were Lenvatinib plus Pembrolizumab (17 studies; n=1996), Avelumab plus Axitinib (5 studies; n=578) and Nivolumab plus Cabozantinib (5 studies; n =484).

**Table 1:** Included studies in Meta-analysis of Immunotherapy and Targeted Therapy Combination Studies.

	No. of studies	No. of participants
**Included Studies**	72	5860
**Abstract/Conference proceedings only**	28% (20/72)	18.7% (1098/5860)
Study Design		
Phase 1b	27.7% (20/72)	16.3% (961/5860)
Phase 2	58.3% (42/72)	32.1% (1886/5860)
Phase 3	13.8% (10/72)	51.4% (3013/5860)
Organ System/Tumour Histology		
**GU**	**22.2% (16/72)**	**37/2% (2182/5860)**
RCC	13.8% (10/72)	32.3% (1898/5860)
Urothelial	5.6% (4/72)	2.6% (152/5860)
Prostate	1.4% (1/72)	7.1% (132/5860)
**Thoracic**	**13.8% (10/72)**	**9.9% (583/5860)**
NSCLC	11.1% (8/72)	8.7% (513/5860)
Thymoma	1.4% (1/72)	0.5% (32/5860)
Mesothelioma	1.4% (1/72))	0.6% (38/5860)
**Hepatobiliary**	13.8% (10/72)	7.4% (434/5860)
HCC	8.3% (6/72)	4.8% (284/5860)
Biliary	5.6% (4/72)	2.6% (150/5860)
**Upper/Lower GI**	**12.5% (9/72)**	**9.8% (577/5860)**
Colorectal	6.9% (5/72)	6.2% (362/5860)
Gastric/oesophageal	5.6% (4/72)	3.7% (215/5860)
Skin		
Melanoma	8.3% (6/72)	14.1% (829/5860)
**Head and Neck**	**6.9% (5/72)**	**1.7% (105/5860)**
Head/Neck SCC	4.1% (3/72)	1.0% (67/5860)
Anaplastic thyroid carcinoma	1.4% (1/72)	0.3% (16/5860)
Adenoid cystic carcinoma	1.4% (1/72)	0.04% (28/5860)
**Gynae**	**9.7% (7/72)**	**15.6% (913/5860)**
Cervical	2.8% (2/72)	14.8% (87/5860
Ovarian	4.2% (2/72)	1.1% (65/5860)
Endometrial	4.2% (3/72)	12.9% (761/5860)
Other		
Breast	6.9% (5/72)	0.1% (49/5860)
GBM	1.4% (1/72)	0.1% (52/5860)
Sarcoma	4.1% (3/72)	2.2% (128/5860)
Multiple tumour types	1.4% (1/72)	2.3% (137/5860)
Therapy		
ICI Target		
PD-L1	34.7% (n=25/72)	34.7% (2034/5860)
PD-1	65.2% (n=47/72)	65.2% (3826/5860)
CTLA-4	1.4% (n=1/72)	0.1% (33/5860)
TKI Target		
Multitargeted TKI	73.6% (52/72)	65% (3801/5860)
VEGF specific	12.5% (9/72)	18.7% (1095/5860)
BRAF & MEKi	9.7% (7/72)	12.3% (726/5860)
MEKi alone	1.4% (1/72)	3.1% (183/5860)
EGFR	2.8% (2/72)	0.01% (42/5860)
ALK	1.4% (1/72)	<0.01% (13/5860)
Most common regimens		
Lenvatinib/Pembrolizumab	23.6% (17/72)	34% (1996/5860)
Avelumab/Axitinib	6.9% (5/72)	9.9% (578/5860)
Nivolumab/Cabozantinib	6.9% (5/72)	3.1% (484/586)

TKI, Tyrosine Kinase Inhibitor; ICI, Immune checkpoint inhibitor; RCC, Renal cell carcinoma; NSCLC, Non-small cell lung cancer; HCC, Hepatocellular carcinoma; SCC, Squamous cell carcinoma; GBM, Glioblastoma Multiforme; PD-L1, Programme Death Ligand 1; CTLA-4, Cytotoxic T Lymphocyte Antigen 4; MEK, Mitogen activated protein kinase BRAF, B-Raf proto oncogene; EGFR, Epidermal Growth Factor Receptor; ALK, Anaplastic Lymphoma Kinase; VEGF, Vascular Endothelial Growth Factor ReceptorBold values indicates the overall toxicity g3-g5 and irAEs.

The majority of the included studies were early phase (Phase Ib or II = 62/72, 87%) and the majority of studies included patients with metastatic disease (n_studies_ = 70/72, 97.2%). The most common tumour types in these studies (see **Table 1**) included RCC (10 studies, n_patients_=1898) melanoma (n_studies_ = 6, n_patients_ = 829), NSCLC (n_studies_ = 8, n_patients_ = 513) and HPB cancers (n_studies_ = 10, n_patients_ = 434).

### Primary analysis

**Figure 2:**
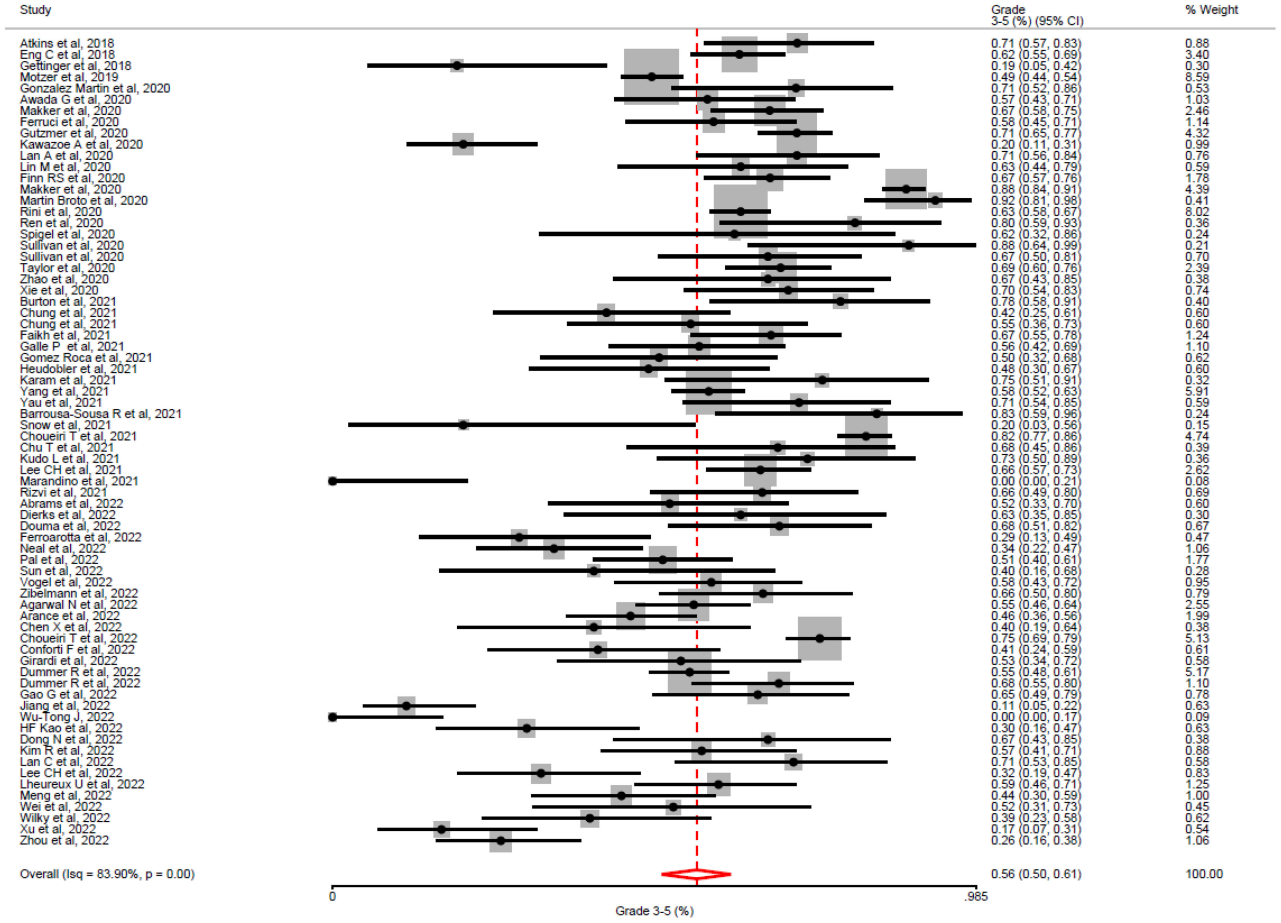
Overall incidence of grade 3-5 toxicity by Common toxicity Criteria for Adverse Events, in all included studies. In all studies utilising TKI-ICI combination, the overall incidence of grade 3 -5 toxicity was 56% (95% CI = 50–61%) with significant heterogeneity between studies (i^2^= 83.9%, p <0.01).

All studies reported the incidence of G3-5 toxicity and were included in our primary analysis. The duration of treatment exposure was reported in 64% (46/72) of studies, with a median duration of 6.2 months and an IQR of 3-9.2 months. In a random-effects model meta-analysis, the overall incidence of G3-5 toxicity (See **Figure 2**) was 56% (95% CI = 50% – 61%). In all included studies, the incidence of G3-5 toxicity ranged from 7 – 92% (IQR: 42 – 68%). We performed a sensitivity analysis (See **Supplementary Appendix**) excluding low-quality studies (CAT score <20, n_studies_= 20/72, 27.7%), and a similar incidence of G3-5 toxicity was demonstrated across remaining studies (53.1%, 95% CI 45.0% – 61.0%). Significant heterogeneity was observed across studies (I^2^= 83.9%; H^2^= 2.65; Q = 193.92, p <0.01). There was no evidence from the meta-regression analysis that primary tumour subgroup (χ^2^= 14.07, p = 0.08), TKI target (χ^2^= 0.34, p = 0.84) or ICI target (χ^2^= 0.14, p = 0.7) accounted for the heterogeneity observed (See **Supplementary Appendix** for meta-regression analysis covariates). This was reported in the majority of studies (63/72, 87.5%). The overall incidence of discontinuation of ICI/TKI combinations due to toxicity was 16% (953/5218), based on reporting in 87.5% of studies (63/72). The overall incidence of G5 toxicity was 2% (95/4740), based on reporting in 77% (56/72) studies.

### Toxicities of interest and irAEs

In pre-defined critical toxicities of interest (See **Table 2**), we report the incidences of: diarrhoea (7.1%, 393/5526); fatigue (4.1%, 213/5106); shortness of breath (1.4%, 34/2269); rash (3.6%, 129/4695); Alanine aminotransferase (ALT) increase (6.6%, 287/5088), Aspartate aminotransferase (AST) increase (5.7%, 236/4972). Notably, the incidence of G3-5 hypertension in all studies was 21.7% (935/4498), consistent with the significant number of patients in this analysis having received VEGF-targeted TKIs. There was significant variation in the missing data in specific symptom toxicity ranging from 5% – 62%.

**Table 2:** Summary of toxicity in common diseases and regimens.

	All	RCC	NSCLC	HPB	Len/Pembro	Ave/Axi	Nivo/Cabo	ICI + Oncogene targeting TKI (BRAF/MEK/EGFR/ALK)
No. of studies	72	10	8	10	**17**	**5**	**5**	**11**
n=	5860	1898	513	434	1996	578	484	964
G3-5 Toxicity**	**54% (95% CI = 49-60%)**	**58% (95% CI = 40% -73%)**	**57% (95% CI = 43-69%)**	**57% (95% CI = 47 -66%)**	**56% (95% CI 43 – 68%)**	**49% (95% CI 39% - 69%)**	**61% (95% CI 44% - 75%)**	**62% (95% CI 53% - 71%)**
Diarrhoea G3-5	7.1% (393/5226)	11.3% (216/1898)	5.6% (9/161)	6.0% (23/383)	9.9% (152/1521)	4.4% (26/578)	6.6% (32/484)	4.8% (47/964)
Fatigue G3-5	4.1% (213/5106)	3.3% (62/1860)	2.5% (4/161)	3.6% (12/333)	3.9% (60/1505)	3.6% (21/578)	3.9% (19/484)	2.5% (23/904)
SOB G3-5	1.4% (34/2269)	1.4% (8/585)	5.1% (3/59)	9.3% (3/32)	1.6% (9/567)	1.4% (8/556)	2.1% (2/95)	2.2% (12/540)
Rash G3-5	2.7% (129/4695)	11.2% (22/1962)	3.9% (8/204)	<1% (3/400)	<0.1% (4/1216)	<0.1% (3/578)	1.5% (6/400)	7.1% (68/964)
ALT increase G3-5	5.6% (287/5088)	6.3% (120/1898)	7.3% (15/204)	8.5% (30/353)	0.2% (21/1254)	3.9% (23/578)	6.1% (30/484)	5.6% (54/964)
AST increase G3-5	4.7% (236/4972)	3.4% (68/1962)	7.3% (15/204)	14.4% (51/353)	2% (29/1254)	2.2% (13/578)	11.6% (56/484)	6.3% (61/964)
Hypertension G3-5	20.7% (935/4498)	21.1% (392/1854)	7% (12/162)	18.2% (79/434)	18% (258/1519)	22.4% (130/578)	11.5% (56/484)	1.9% (6/312)
**irAE Toxicity G3-5**	**12.8% (390/3042)**	**12.9%** **(231/1787)**	**20.2% (18/89)**	**28.4% (25/88)**	**2.3% (18/759)**	**11.0% (49/444)**	**14.2% (49/343)**	**41% (33/81)**
irAE Colitis G3-G5	1.4% (42/2830)	1.7% (24/1345)	0% (0/68)	1.7% (1/57)	<0.1% (1/570)	0% (0/76)	1.4% (5/343)	1.4% (6/439)
irAE Hepatitis G3 – G5	2.1% (52/2470)	2.0% (27/1345)	4.5% (4/89)	14.0% (8/57)	1.1% (8/673)	1.3% (1/76)	2.6% (9/343)	1.1% (3/264)
IrAE Pneumonitis G3-G5	1.1% (38/3471)	1% (14/1345)	5.3% (7/132)	4.4% (4/89)	<0.1% (5/736)	1.8% (2/108)	<0.1% (3/343)	2.7% (8/295)

NSCLC, Non-small cell lung cancer; RCC, Renal cell carcinoma; HPB, Hepatobiliary cancers; ICI, Immunecheckpoint inhibitors.

Len/Pembro, Lenvatinib + Pembrolizumab; Ave/Axi, Avelumab + Axitinib; Nivo/Cabo, Nivolumab + Cabozantinib.

BRAF, B-raf; MEK, Mitogen-activated protein kinase; EGFR, Epidermal like growth factor receptor; ALK, Anaplastic Lymphoma Kinase.

**No missing data for overall incidence of G3-5 toxicity across studies but missing data for individual toxicities & ICIs varied by disease type and toxicity – hence different total numbers for each participant group**.Bold values indicates the overall toxicity g3-g5 and irAEs.

There was unfortunately limited reporting on irAEs in included studies (See **Table 2**) with almost 50% of data missing on the overall incidence of G3-5 irAEs (2818/5860, 48.0%). In those studies in which the incidence and spectrum of irAEs was specifically annotated, the incidence of G3-5 ICI-associated toxicity was 12.8% (390/3042) – consistent with experience in the ICI monotherapy setting. The incidence of critical toxicities of G3-5 colitis, hepatitis and pneumonitis was 1.4% (42/3820), 2.1% (52/2470) and 1.1% (38/3471), respectively.

### Toxicity by primary tumour site

#### Non-small cell lung cancer

**Figure 3:**
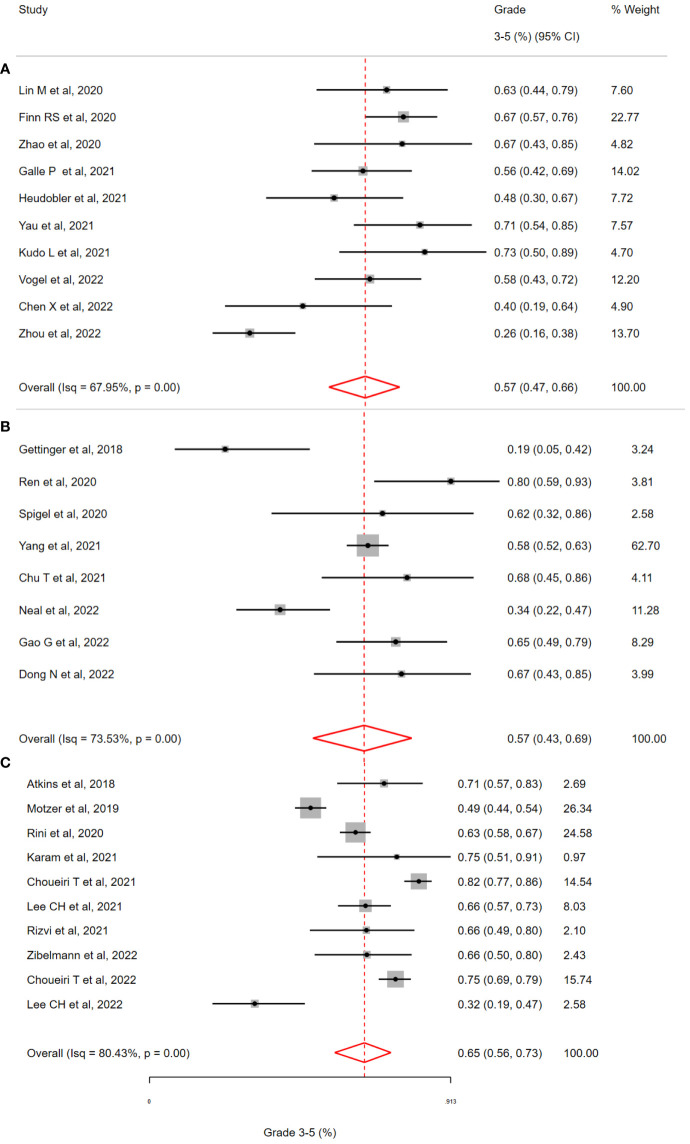
Incidence of grade 3-5 toxicity in selected clinical trials of patients with **(A)** Hepatobiliary Cancers **(B)** Non-small cell lung cancer **(C)** Renal cell cancer, treated with Immune Checkpoint Inhibitor and Targeted Therapy Combinations. **(A)** The overall incidence of grade 3-5 toxicity in patients with hepatobiliary (HCC & Biliary) cancer was 57 % (95% CI 47-66%, i^2^=68.0%). **(B)** The overall incidence of grade 3-5 toxicity in patients with non-small cell lung cancer was 57 % (95% CI 43-69%, i^2^=73.5%). **(C)** The overall incidence of grade 3-5 toxicity in patients with renal cell cancer was 65% (95% CI 56-73%, i^2^=80.4%).

For patients with NSCLC (See **Figure 3**), most studies (5/8, 63%) involved treatment with a PD-(L)1 inhibitor and a multitargeted TKI (Targeting *VEGF-1* and others). The three remaining studies involved TKIs targeting oncogenes [Erlotinib (*EGFR*), Alectinib & Crizotinib (*ALK*)]. The pooled incidence of grade 3-5 toxicity was 57% (95% CI = 43-69%;I^2^= 73.53%; H^2^= 1.04; Q = 8.23, p <0.01). The randomised phase III study of Lenvatinib plus pembrolizumab contributed the most patients (309/513) to the NSCLC subgroup. This study remains unpublished at the time of review and detailed data on adverse event reporting is unavailable. For this reason, when focusing on specific symptoms and irAEs, the analysis was limited by missing data (74.3%, 381/513). For patients receiving oncogene-targeting TKIs, a high burden of toxicities was observed in included studies (n_studies_ =3). This included severe hepatic toxicities - two patient deaths occurred with the combination of nivolumab plus crizotinib, and an incidence of G3-5 toxicity of 66.6% for patients receiving alectinib plus atezolizumab. In the NSCLC cohort, studies by *Gettinger et al.* (Nivolumab plus Erlotinib in *EGFR*-mutant NSCLC) and *Neal et al.* (Atezolizumab plus Cabozantinib had the lowest rates of G3-5 toxicity reported at 19% and 34% respectively (17, 18). Both of these studies (63% of patients, 50/79) included patients with *EGFR*-mutant lung cancer, unlike any other studies in the NSCLC cohort.

#### Renal cell carcinoma

In studies of RCC (n=10, see **Figure 3**), four randomised phase III studies (CLEAR, Checkmate9ER, Javelin Renal101, KEYNOTE-426) contributed significantly to the patients receiving ICI/TKI (n=1898). The pooled incidence of G3-5 toxicity was 58% (95% CI 40-73%) with observed heterogeneity (I^2^= 80.43%; H^2^= 3.52; Q = 30.19, p <0.01) All of these studies involved combining a PD-(L)1 inhibitor with a multi-targeted TKI (e.g. Cabozantinib) or a VEGF-specific TKI (e.g. Axitinib). Consistent with the overall population (all studies), G3-5 hypertension accounted for a significant burden of the G3-5 toxicity (392/1854, 21.1%). The overall incidence of G3-5 irAEs was 12.9%, based on reporting in >90% of patients (94.1%, 1787/1898). The incidence of G3-5 irAEs associated colitis, hepatitis and pneumonitis was low (1.7%, 2.0% and 1.0% respectively). The reported incidence of G3-5 toxicity was lowest in two studies; *Motzer* et al. (Avelumab plus Axitinib in RCC, G3-5 Toxicity of 49%) and *Lee CH* et al. (Lenvatinib plus Pembrolizumab in non-clear cell RCC, G3-5 Toxicity of 32%). This was the only study which included non-clear cell RCC in the cohort. The low toxicity observed with the combination of Avelumab plus Axitinib is consistent with this profile of this regimen across disease types (See section below: Toxicity by Specific TKI/ICI combination regimen).

#### Hepatobiliary cancers

For patients with hepatobiliary cancers (HCC = 6, BTC = 4, see **Figure 3**), a total of 434 patients received therapy with an ICI/TKI combination (See **Table 2**). In these studies, all patients received a PD-(L)1 inhibitor with a multitargeted TKI (except for one treated with Axitinib and another study evaluating a PD-1/CTLA-4 combination). The overall incidence of G3-5 toxicity in this group was 57% (95% CI = 47% – 66%) with heterogeneity observed (I^2^= 67.95%; H^2^= 1.28; Q = 9.78, p <0.01). Specific toxicities were reported in >80% (81.3%, 353/434) of patients except in the case of shortness of breath which was reported in <10% (7.3%, 32/434) of patients. The incidence of AST/ALT elevation was numerically higher for patients with HPB cancers than for all patients (ALT: 8.5% versus 5.6%; AST: 14.4% versus 4.7%). The incidence of irAEs was reported in just 20.2% of patients (88/434) A significant number of those patients (64.7%, n=57/88) received combination PD-1/CTLA-4 inhibition (the only study of 72 with this combination), thus comparison with other groups is limited. Studies by *Chen et al.* (Sintilimab plus Anlotinib in HCC) and *Zhou et al.* (Anlotinib plus TQB2450) demonstrated the lowest G3-5 toxicity of 40% and 26%, respectively (19, 20) et al. (Sintilimab plus Anlotinib in HCC). These two studies were the only trials in which patients received the TKI Anlotinib.

### Toxicity by specific TKI/ICI combination regimen

**Figure 4:**
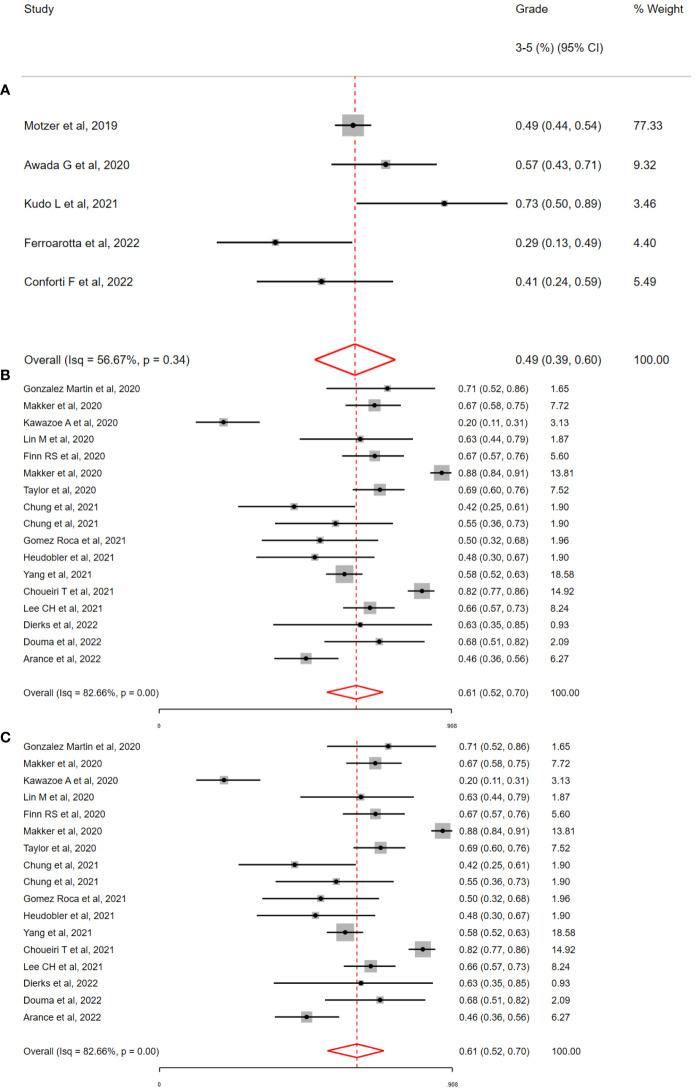
Incidence of grade 3-5 toxicity by immunotherapy and tyrosine kinase inhibitor regimen, by **(A)** Avelumab & Axitinib (A/A) **(B)** Lenvatinib & Pembrolizumab (L/P) **(C)** Nivolumab & Cabozantinib (N/C). The overall incidence of grade 3-5 toxicity was high with all three combinations; 49% with A/A, 56% with L/P and 61% with N/C. Significant interstudy heterogeneity was observed with the combination of L/P & N/C but not with A/A (i^2^=56%, p =0.37).

Based on available studies (>5) and total patients (>450), three regimens (See **Figure 4**) were selected for subgroup meta-analysis (Lenvatinib/Pembrolizumab, L/P; Avelumab/Axitinib, A/A; Nivolumab/Cabozantinib, N/C). Across these studies, the overall incidence of G3-5 toxicity was consistent with the overall population (L/P = 56% 95% CI = 43-68%; A/A = 49% 95% CI = 39%-69%; N/C = 61% 95% CI 44%-75%). Despite receiving the same drug therapies, significant heterogeneity was observed in G3-5 toxicity associated with these regimens (L/P, I^2^= 82.66%, H^2^= 5.37; Q = 103.89, p <0.01; N/C, I^2^= 79.71%, H^2^= 2.21; Q = 9.10, p <0.01) with the exception of A/A (I^2^= 56.67%, H^2^= 1.0; Q = 3.01, p =0.34). For patients receiving L/P (See **Table 2**), the most commonly recorded G3-5 toxicities included diarrhoea (9.9%, 152/1521) and hypertension (16.9%, 258/1519). A/A was associated with the lowest overall G3-5 toxicities with hypertension the most commonly reported (22.5%, 130/578). Hypertension was numerically lower in the N/C cohort (14.2%, 49/343). In the L/P cohort, the incidence of G3-5 irAEs was low (2.3%, 18/759). However, >50% (61.9%, 1237/1996) of patient data was not reported in this cohort so these results may underestimate this toxicity. The incidence of G3-5 ICI irAEs was 11.0% (49/444) and 14.3% (49/343) in the A/A & N/C cohorts respectively.

For patients who received ICI with TKIs targeting specific oncogenes (*BRAF, MEK, EGFR, ALK*; n_studies_ = 11), the incidence of G3-5 toxicity ranged from 18% to 88% (Interquartile range = 58% - 71%). In a random effects meta-analysis, the overall incidence of G3-5 toxicity was 61% (95% CI = 53% - 71%;I^2^ = 62.19%, H^2^= 1.09; Q = 10.71, p <0.01). The most common G3-5 toxicities included diarrhoea 4.8% (47/964), Rash 7.1% (68/964), AST increase 6.3% (61/964), ALT increase 5.6% (54/964) with less common toxicities of Fatigue 2.5% (23/904); SOB 2.2% (12/540); Hypertension 1.9% (6/312). Notably but not unexpectedly, the overall incidence of G3-5 hypertension was low at 1.9% (6/312). This is in contrast to the overall study population where hypertension occurred in 21.1% of patients treated with TKI-ICI combination therapy. Notably, the incidence of rash was higher (7.1% with oncogene targeted TKIs versus 2.7% in the overall study population). The incidence of irAEs was also higher (41.8% with oncogene targeting TKIs versus 12.8% in the overall study population), although small numbers in the oncogene group may confound results.

### Sequential TKI followed by ICI (‘Run-in’ period)

**Figure 5:**
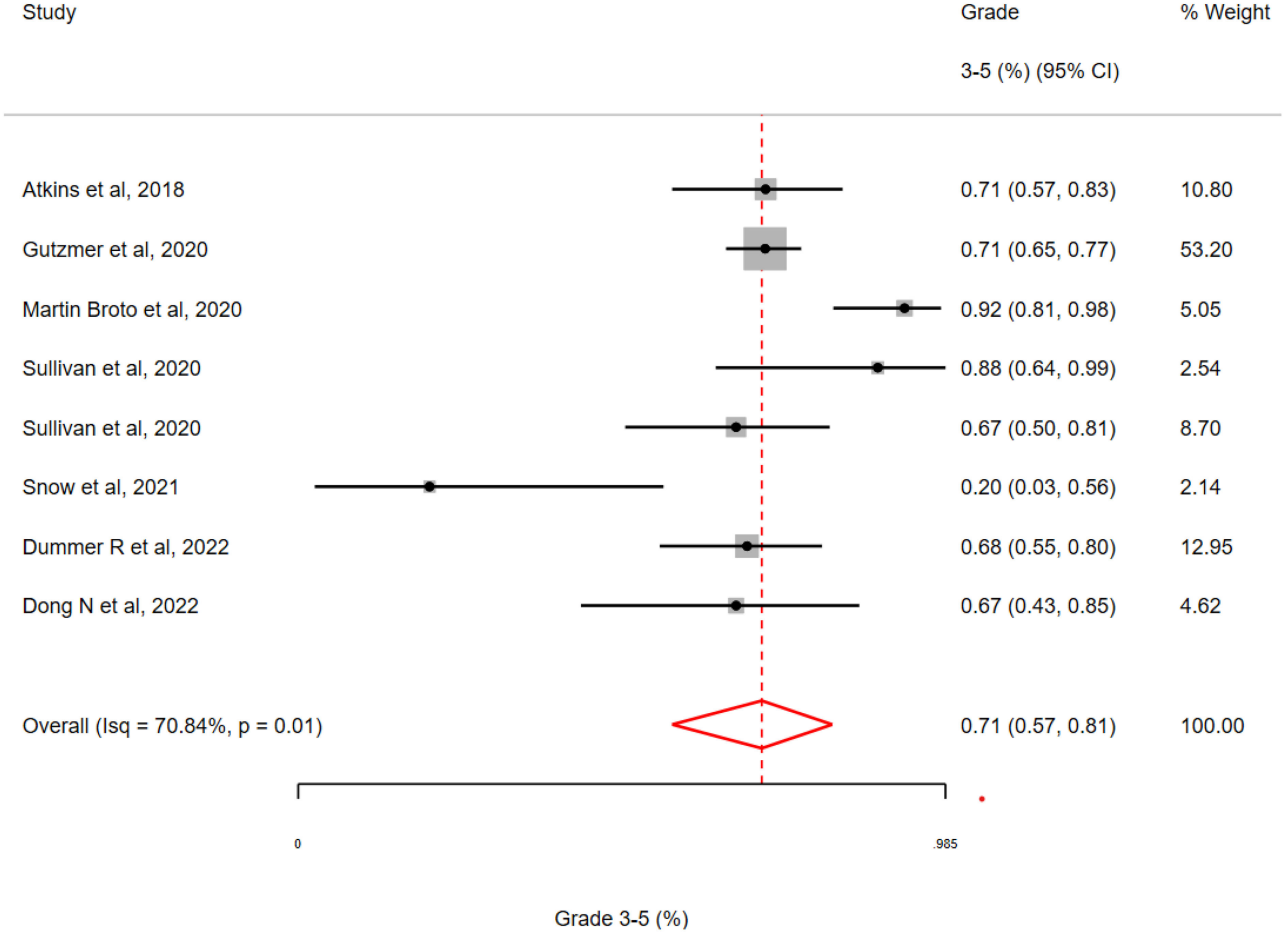
Incidence of grade 3-5 toxicity in clinical trials with a ‘run-in’ period involving a targeted therapy followed by an immune checkpoint inhibitor. The pooled estimate of grade 3-5 toxicity was 71% for all studies with a range of 20 – 92% (Interquartile Range 67-73%.

A total of 8 studies with a run-in period of between 1 and 4 weeks were included. These studies included a run-in with Vemurafenib (n=1), Cobimetanib plus Vemurafenib (n=3), Alectinib (n=1), Sunitinib (n=1), Axitinib (n=1) or Sitravatinib (n=1). The overall toxicity of studies employing a TKI-run-in was 71% (95% CI 57-81%;I^2^ = 70.84%, H^2 =^ 1.00; Q = 5.57, p =0.01) with just one study reporting an overall incidence of G3-5 toxicity of <60% (See **Figure 5**).

## Discussion

Combining ICIs with TKIs represents an opportunity for therapeutic synergy to improve outcomes from ICIs alone, yet can be associated with high-grade toxicity. In this comprehensive meta-analysis of 72 Phase Ib – III studies of TKI/ICI combinations across solid tumour types, the observed overall incidence of G3-5 toxicity (56%) was apparent across subgroups of the meta-analysis by tumour type (NSCLC, RCC, HPB) and treatment regimen (Lenvatinib plus Pembrolizumab, Avelumab plus Axitinib, Nivolumab plus Cabozantinib). Although the overall burden of toxicity was similar with different regimens, the incidence of specific toxicities observed varied with different TKI partners (*VEGF*/Multitargeted; Hypertension; Oncogene targeted TKIs; Rash, irAEs) and there was a high degree of heterogeneity between studies. Finally, while a TKI run-in strategy has been purported to potentially mitigate the toxicity of TKI/ICI combinations, a high incidence of G3-5 toxicity was observed in our included studies.

Our data suggests that the incidence of irAEs is higher for patients who receive oncogene targeting TKIs versus multitargeted/VEGF specific TKIs. In a retrospective analysis of patients who received the TKI, Sotorasib, the incidence of G3-5 toxicity was significantly higher in the group which had received an ICI in the preceding 30 days (33% versus 11%) (21). Specifically, it has been described that sotorasib in combination/sequential with PD-(L)1 inhibition is associated with a high-incidence of immune mediated hepatotoxicity (10, 21). Similarly, this has been demonstrated with the combination of crizotinib plus PD(L)-1 inhibition (22). In pre-clinical models, treatment with sotorasib has been shown to induce a pro-inflammatory tumour microenvironment, which may contribute to the synergistic toxicity of ICI/TKI combinations (23). A similar effect of the tumour immune microenvironment has been demonstrated with alectinib and osimertinib (24, 25). Our data supports this potential shared mechanism across oncogene-targeted TKIs which may underlie the high incidence of irAEs with these combinations.

A significant challenge in interpretation of irAE events in our study is the often absent reporting. The FDA recommends reporting of all irAEs including the duration, outcome, therapy if commenced and duration of irAE (26). There was very limited adherence to these guidelines in our included studies. A clinical challenge may be the differentiation of an irAE versus an AE for patients receiving combination therapy (eg ICI/TKI combination). Attention to this challenge is of increasing relevance with increased use of ICIs in combination with TKIs, chemotherapies and antibody drug conjugates (ADCs). The limited data presented in combination studies with regards to irAEs impairs clinicians and patients ability to make informed treatment decisions with the best available evidence. Our study contributes an analysis of ICI/TKI combination studies which raises a concerning trend of limited reporting of irAEs. A framework is needed to guide investigators in determining the aetiology of an AE and to ensure comprehensive reporting.

Our data would suggest that the studies with a TKI run-in were associated with what is generally considered an unacceptable incidence of G3-5 toxicity (G3-5 toxicity = 71%). Atezolizumab plus Vemurafenib was one of the earliest combinations explored in a run-in strategy (27). However, this was modified after only 3 initial patients experienced G3 toxicity. In a retrospective analysis of patients who received Osimertinib before or after PD-(L)1 inhibition, the authors discovered a high incidence of severe irAEs when PD-(L)1 inhibition was followed by Osimertinib (within 20 days) but not when Osimertinib was followed by PD-(L)1 (25). Our data contributes prospective data to the run-in approach and suggests that a run-in does not consistently mitigate the overall incidence of G3-5 toxicity. However, to conclusively address this question, randomised data would be needed in specific disease and treatment settings.

Our work has several limitations. Firstly, the studies included were associated with clinical heterogeneity which limits our interpretation of the pooled estimates, meaning our results are exploratory and hypothesis generating. However, we included different ICI/TKI combinations and diseases to ensure a comprehensive review. Despite our broad inclusion criteria, all studies involved an ICI targeting PD-1/PD-L1 and the vast majority of studies included a TKI targeting VEGF (VEGF specific or multitargeted; 85%, 61/72). We also saw in our work that there is significant heterogeneity in the incidence of G3-5 toxicity even when we focused on one regimen (eg Lenvatinib and Pembrolizumab). This is also apparent in other published works which focus on PD-1/PD-L1 inhibitors alone, where significant heterogeneity was also observed (28, 29). Our and others work are indicative that heterogeneity observed in toxicity meta-analysis may occur even when ICI/TKI combinations and diseases are homogenous. This supports our conclusions that further work is needed to harmonise reporting of AEs, which may contribute to observed heterogeneity. Nonetheless, the clinical heterogeneity and statistical heterogeneity observed is a limitation. Potential bias that may have reflected our results include publication bias. Given that we were assessing toxicity, rather than efficacy, it was our expectation that publication bias would be less likely to be a significant counfounder. Potential other biases include search and selection bias. We mitigated these biases with the designing of a search strategy with an information specialist and multiple reviewers of abstracts/full-texts. Furthermore, the exclusion of phase 1a studies (to avoid dose-finding studies) resulted in a significant number of excluded studies which may have had clinically relevant results (30–40). We did not have access to patient-level data, therefore results we included were limited to that available in publications or conference proceedings. This resulted in missing data (Range of 10.5% - 61.5%) which may have introduced bias to our symptom specific (e.g. diarrhoea) and toxicity specific (eg irAE) data. We were not able to provide an overall incidence of toxicity in a ‘Control Arm’ in our work given that the majority of included studies were not randomised and those that were may have had a control arm which did not include either therapy in the investigational arm (eg Checkmate9ER – Nivolumab & Cabozantinib versus Sunitinib) (41). If included studies included a control arm, it would have allowed us to compare relatively toxicity across studies potentially mitigating some of the challenges observed with heterogenous results. Studies which closed early due to excess toxicity are less likely to be published and as such there may be a reporting bias in included studies. Finally, the inclusion of studies which have not been peer-reviewed but data available as conference proceedings is a limitation but was intended to ensure a broad group of studies was included.

In conclusion, this study aims to address the underrepresented topic of the nuances of toxicity of ICI/TKI combinations - a growing set of oncology regimens used across tumours - in the immunotherapy armamentarium. These data are in fact critical to clinical decision-making, particularly when multiple treatment options exist- and when toxicity becomes a key deciding factor when clinicians select appropriate therapy in partnership with patients. We have identified that more than half of patients receiving these therapies will experience a diverse range of G3-5 toxicity which does not appear to be mitigated by a run-in strategy. Reporting of irAEs in ICI/TKI studies is limited and a framework is needed to ensure adequate reporting of incidence, duration and treatment of AEs in studies. Future directions to compliment comprehensive reporting may include use of patient reported outcomes, collection of financial and time toxicity data, and novel clinical trial designs employing metronomic dosing, and other toxicity mitigation approaches.

## Data availability statement

The raw data supporting the conclusions of this article will be made available by the authors, without undue reservation.

## Author contributions

DO’R: Data curation, Formal analysis, Investigation, Methodology, Project administration, Software, Writing – original draft, Writing – review & editing. CO’L: Formal analysis, Writing – original draft, Writing – review & editing. AR: Formal analysis, Writing – original draft, Writing – review & editing. MT: Formal analysis, Methodology, Writing – original draft, Writing – review & editing. GO’K: Writing – original draft, Writing – review & editing. LH: Formal analysis, Methodology, Writing – original draft, Writing – review & editing. KB: Formal analysis, Methodology, Writing – original draft, Writing – review & editing. JN: Conceptualization, Data curation, Formal analysis, Methodology, Supervision, Validation, Writing – original draft, Writing – review & editing.
