# Supplementary material for: Toxicity of immune checkpoint inhibitors and tyrosine kinase inhibitor combinations in solid tumours: a systematic review and meta-analysis
Source: Front Oncol. 2024 Jul 15;14:1380453. doi: 10.3389/fonc.2024.1380453 (PMC11284079; doi:10.3389/fonc.2024.1380453)
Supplement: Supplementary file 1 [file DataSheet_1.docx]

**Supplementary Data Appendix**

**
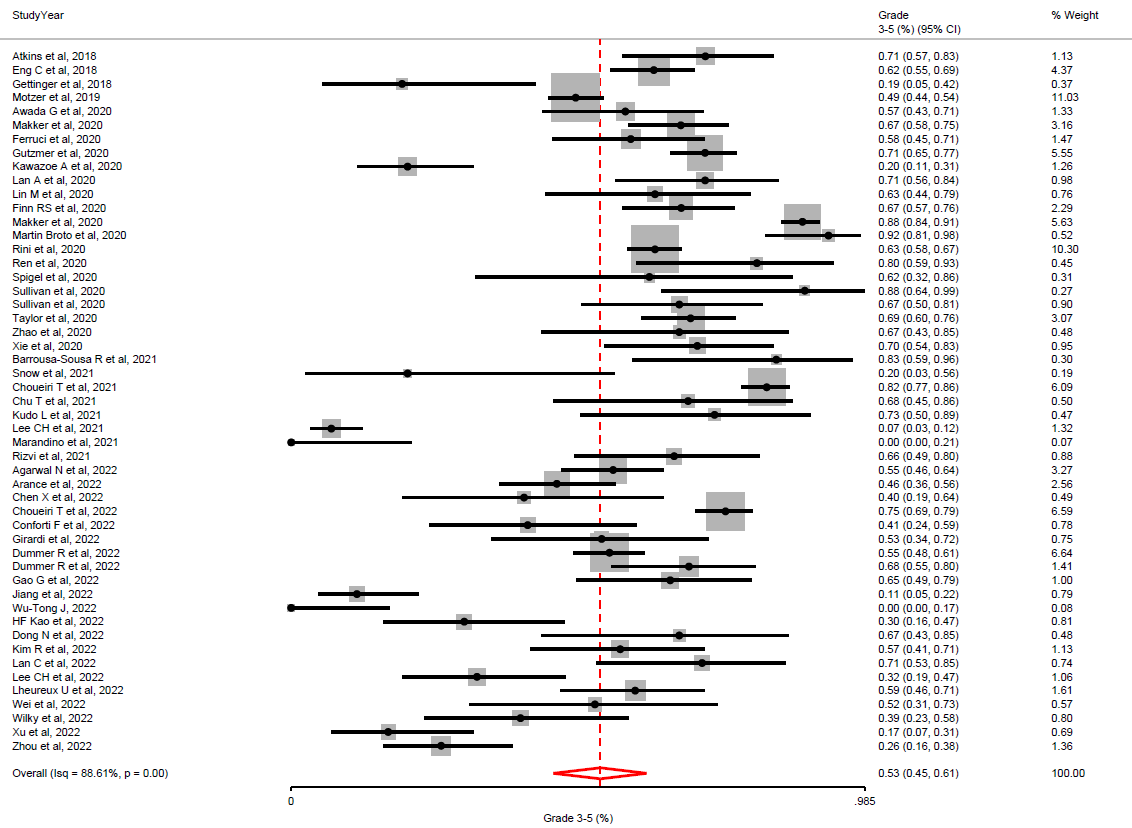
**

**Supplementary Figure 1 – Overall incidence of G3-5 toxicity with low quality (Crowe Critical Appraisal Tool <20) studies excluded**

| **Short Title** | **Citation** | **n =** | **Drug**  **With**  **Run-in** | **Run-in time (weeks)** | **G3-5 Toxicity** |
| --- | --- | --- | --- | --- | --- |
| Atezolizumab/cobimetinib/vemurafenib in BRAF mutant melanoma | Sullivan et al, 2020 | 17 | Vemurafenib | 1 | 88% |
| Atezolizumab/cobimetinib/vemurafenib in BRAF mutant melanoma | Sullivan et al, 2020 | 39 | Cobimetinib & Vemurafenib | 4 | 66% |
| Atezo/vemurafenib/cobimetinib in patients with melanoma with CNS metastases | Dummer et al, 2022 | 60 | Cobimetinib & Vemurafenib | 3 | 68% |
| Atezolizumab, vemurafenib, and cobimetinib in advanced melanoma (IMspire150) | Gutzmer et al, 2020 | 514 | Cobimetinib & Vemurafenib | 4 | 35% |
| Alectinib Plus Atezolizumab in Advanced ALK Rearranged NSCLC | Dong et al, 2022 | 21 | Alectinib | 1 | 66% |
| Nivolumab and sunitinib in advanced soft tissue sarcoma | Martin Broto et al, 2022 | 52 | Sunitinib | 1 | 92% |
| Axitinib in combination with pembrolizumab in RCC | Atkins et al, 2018 | 52 | Axitinib | 1 | 71% |
| Sitravatinib and nivolumab for resectable Oral SCC | Snow et al, 2021 | 10 | Sitravatinib | . | 20% |

**Supplement Table 1 – Summary of studies with run in of TKI**

**
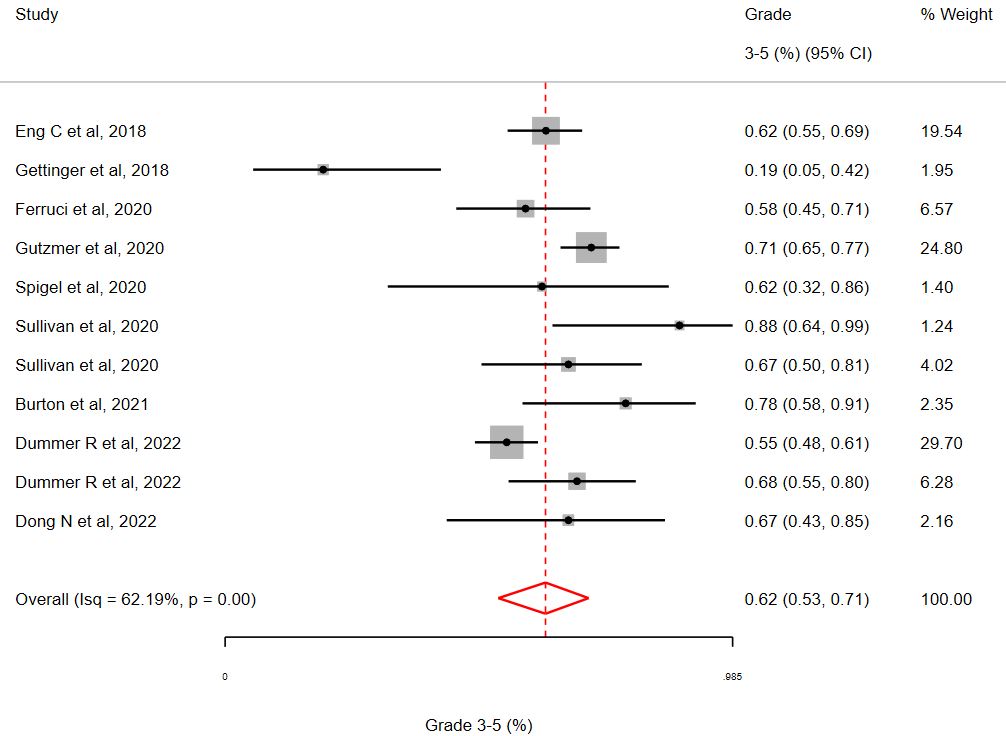
**

**Supplementary Figure 2 – Overall incidence of G3-5 toxicity when ICI combined with TKI targeting oncogene (BRAF/MEK/EGFR/ALK)**

| Abrams, T. A., Kazmi, S. M. A., Seth Winer, I., Subbiah, V., Falchook, G. S., Reilley, M., Kunk, P. R., Goel, S., Garrido-Laguna, I., Kochenderfer, M. D., Werneke, S., Andrianova, L., Sudhagoni, R., & Paulson, S. (2022). A phase 1b multitumor cohort study of cabozantinib plus atezolizumab in advanced solid tumors (COSMIC-021): Results of the colorectal cancer cohort. *Journal of Clinical Oncology*, *40*(4 SUPPL). https://doi.org/10.1200/JCO.2022.40.4-suppl.121 |
| --- |
| Agarwal, N., Loriot, Y., McGregor, B. A., Dreicer, R., Dorff, T. B., Maughan, B. L., Kelly, W. K., Pagliaro, L. C., Srinivas, S., Squillante, C. M., Vaishampayan, U. N., Liu, Y., Curran, D., Choueiri, T. K., & Pal, S. K. (2020). Cabozantinib (C) in combination with atezolizumab (A) in patients (pts) with metastatic castration-resistant prostate cancer (mCRPC): Results of Cohort 6 of the COSMIC-021 Study. *Journal of Clinical Oncology*, *38*(6_suppl), 139. https://doi.org/10.1200/JCO.2020.38.6_suppl.139 |
| Arance, A. M., De la Cruz-Merino, L., Petrella, T. M., Jamal, R., Ny, L., Carneiro, A., Berrocal, A., Marquez-Rodas, I., Spreafico, A., Atkinson, V., Svedman, F. C., Mant, A., Smith, A. D., Chen, K., Diede, S. J., Krepler, C., & Long, G. V. (2021). Lenvatinib (len) plus pembrolizumab (pembro) for patients (pts) with advanced melanoma and confirmed progression on a PD-1 or PD-L1 inhibitor: Updated findings of LEAP-004. *Journal of Clinical Oncology*, *39*(15 SUPPL). https://doi.org/10.1200/JCO.2021.39.15_suppl.9504 |
| Atkins, M. B., Plimack, E. R., Puzanov, I., Fishman, M. N., McDermott, D. F., Cho, D. C., Vaishampayan, U., George, S., Olencki, T. E., Tarazi, J. C., Rosbrook, B., Fernandez, K. C., Lechuga, M., & Choueiri, T. K. (2018). Axitinib in combination with pembrolizumab in patients with advanced renal cell cancer: a non-randomised, open-label, dose-finding, and dose-expansion phase 1b trial. *The Lancet. Oncology*, *19*(3), 405–415. https://doi.org/https://dx.doi.org/10.1016/S1470-2045(18)30081-0 |
| Awada, G., Ben Salama, L., De Cremer, J., Schwarze, J. K., Fischbuch, L., Seynaeve, L., Du Four, S., Vanbinst, A.-M., Michotte, A., Everaert, H., Rogiers, A., Theuns, P., Duerinck, J., & Neyns, B. (2020). Axitinib plus avelumab in the treatment of recurrent glioblastoma: a stratified, open-label, single-center phase 2 clinical trial (GliAvAx). *Journal for ImmunoTherapy of Cancer*, *8*(2). https://doi.org/https://dx.doi.org/10.1136/jitc-2020-001146 |
| Barroso-Sousa, R., Keenan, T. E., Li, T., Tayob, N., Trippa, L., Pastorello, R. G., Richardson Iii, E. T., Dillon, D., Amoozgar, Z., Overmoyer, B., Schnitt, S. J., Winer, E. P., Mittendorf, E. A., Van Allen, E., Duda, D. G., & Tolaney, S. M. (2021). Nivolumab in combination with cabozantinib for metastatic triple-negative breast cancer: a phase II and biomarker study. *NPJ Breast Cancer*, *7*(1). https://doi.org/10.1038/s41523-021-00287-9 |
| Burton, E. M., Amaria, R. N., Glitza, I. C., Milton, D. R., Diab, A., Patel, S. P., McQuade, J. L., Honaker, V., Wong, M. K. K., Hwu, P., Wargo, J. A., Davies, M. A., & Tawbi, H. A.-H. (2021). Phase II Study of TRIplet combination Nivolumab (N) with Dabrafenib (D) and Trametinib (T) (TRIDeNT) in patients (pts) with PD-1 naïve or refractory BRAF-mutated metastatic melanoma (MM) with or without active brain metastases. *Journal of Clinical Oncology*, *39*(15_suppl), 9520. https://doi.org/10.1200/JCO.2021.39.15_suppl.9520 |
| Chen, X., Li, W., Wu, X., Zhao, F., Wang, D., Wu, H., Gu, Y., Li, X., Qian, X., Hu, J., Li, C., Xia, Y., Rao, J., Dai, X., Shao, Q., Tang, J., Li, X., & Shu, Y. (2022). Safety and Efficacy of Sintilimab and Anlotinib as First Line Treatment for Advanced Hepatocellular Carcinoma (KEEP-G04): A Single-Arm Phase 2 Study. *Frontiers in Oncology*, *12*, 909035. https://doi.org/https://dx.doi.org/10.3389/fonc.2022.909035 |
| Choueiri, T. K., Eto, M., Kopyltsov, E., Rha, S. Y., Porta, C. G., Motzer, R., Grünwald, V., Hutson, T. E., Méndez-Vidal, M. J., Hong, S. H., Winquist, E., Goh, J. C. H., Maroto, P., Buchler, T., Takagi, T., Rodriguez-Lopez, K., Xing, D., Smith, A. D., & Powles, T. (2021). Phase III CLEAR trial in advanced renal cell carcinoma (aRCC): Outcomes in subgroups and toxicity update. *Annals of Oncology*, *32*, S683–S685. https://doi.org/10.1016/j.annonc.2021.08.056 |
| Choueiri, T. K., Larkin, J. M. G., Oya, M., Thistlethwaite, F. C., Martignoni, M., Nathan, P. D., Powles, T., McDermott, D. F., Robbins, P. B., Chism, D. D., Cho, D. C., Atkins, M. B., Gordon, M. S., Gupta, S., Uemura, H., Tomita, Y., Compagnoni, A., Di Pietro, A., & Rini, B. I. (2017). First-line avelumab + axitinib therapy in patients (pts) with advanced renal cell carcinoma (aRCC): Results from a phase Ib trial. *Journal of Clinical Oncology*, *35*(15). https://www.embase.com/search/results?subaction=viewrecord&id=L617388429&from=export |
| Choueiri, T. K., Powles, T., Burotto, M., Bourlon, M. T., Zurawski, B., Oyervides Juárez, V. M., Hsieh, J. J., Basso, U., Shah, A. Y., Suarez, C., Hamzaj, A., Barrios, C. H., Richardet, M., Pook, D., Tomita, Y., Escudier, B., Zhang, J., Simsek, B., Apolo, A. B., & Motzer, R. J. (2020). Nivolumab + cabozantinib vs sunitinib in first-line treatment for advanced renal cell carcinoma: First results from the randomized phase III CheckMate 9ER trial. *Annals of Oncology*, *31*, S1159-. https://doi.org/10.1016/j.annonc.2020.08.2257 |
| Chu, T., Zhong, R., Zhong, H., Zhang, B., Zhang, W., Shi, C., Qian, J., Zhang, Y., Chang, Q., Zhang, X., Dong, Y., Teng, J., Gao, Z., Qiang, H., Nie, W., Zhao, Y., Han, Y., Chen, Y., & Han, B. (2021). Phase 1b Study of Sintilimab Plus Anlotinib as First-line Therapy in Patients With Advanced NSCLC. *Journal of Thoracic Oncology : Official Publication of the International Association for the Study of Lung Cancer*, *16*(4), 643–652. https://doi.org/https://dx.doi.org/10.1016/j.jtho.2020.11.026 |
| Chung, H. C., Lwin, Z., Gomez-Roca, C., Longo, F., Yanez, E., Alvarez, E. C., Graham, D., Doherty, M., Cassier, P., Lopez, J. S., Basu, B., Hendifar, A. E., Maurice-Dror, C., Gill, S. S., Ghori, R., Kubiak, P., Jin, F., Norwood, K. G., & Saada-Bouzid, E. (2021). LEAP-005: A phase II multicohortstudy of lenvatinib pluspembrolizumab in patients with previously treated selected solidtumors-Results from the gastriccancer cohort. *Journal of Clinical Oncology*, *39*(3 SUPPL). https://doi.org/10.1200/JCO.2021.39.3_suppl.230 |
| Chung, H. C., Saada-Bouzid, E., Munoz, F. L., Yanez, E., Im, S. A., Castanon, E., Graham, D. M., Garcia-Corbacho, J., Lopez, J., Ghori, R., Dutcus, C., Smith, A., Norwood, K., & Gomez-Roca, C. (2021). Lenvatinib plus pembrolizumab forpreviously treated, advanced triple-negative breastcancer: Early results from the multicohort phase 2 LEAP-005 study. *Cancer Research*, *81*(4 SUPPL). https://doi.org/10.1158/1538-7445.SABCS20-PS12-07 |
| Conforti, F., Zucali, P. A., Pala, L., Catania, C., Bagnardi, V., Sala, I., Della Vigna, P., Perrino, M., Zagami, P., Corti, C., Stucchi, S., Barberis, M., Guerini-Rocco, E., Di Venosa, B., De Vincenzo, F., Cordua, N., Santoro, A., Giaccone, G., & Martino De Pas, T. (2022). Avelumab plus axitinib in unresectable or metastatic type B3 thymomas and thymic carcinomas (CAVEATT): a single-arm, multicentre, phase 2 trial. *The Lancet. Oncology*. https://doi.org/https://dx.doi.org/10.1016/S1470-2045(22)00542-3 |
| Da Motta Girardi, D., Niglio, S. A., Mortazavi, A., Lara, P., Pal, S. K., Saraiya, B., Cordes, L. M., Ley, L., Ortiz, O. S., Cadena, J., Diaz, C., Bagheri, M. H., Steinberg, S. M., Costello, R., Streicher, H., Wright, J., Parnes, H. L., Ning, Y. M., Bottaro, D. P., & Apolo, A. B. (2020). Phase I expansion study of cabozantinib plus nivolumab (CaboNivo) in metastatic urothelial carcinoma (mUC) patients (pts) with progressive disease following immune checkpoint inhibitor (ICI) therapy. *Journal of Clinical Oncology*, *38*(15). https://doi.org/10.1200/JCO.2020.38.15_suppl.5037 |
| Dierks, C., Ruf, J., Seufert, J., Klein, C., Kroiss, M., Spitzweg, C., Mayer, P., Kreissl, M., Brandenburg, T., Thomusch, O., Lorenz, K., Zielke, A., & Miething, C. (2021). Lenvatinib/pembrolizumab in metastasized anaplastic thyroid carcinoma (atc): Interim results of the atlep trial. *Thyroid*, *31*(SUPPL 1), A2-. https://doi.org/10.1089/thy.2021.29115.abstracts |
| Douma, L. A. H., de Gooijer, C. J., Noort, V. V. D., Lalezari, F., de Vries, J. F., Vermeulen, M., Schilder, B., Smesseim, I., Baas, P., & Burgers, J. A. (2022). OA04.06 PEMbrolizumab Plus Lenvatinib In Second And Third Line Malignant Pleural MEsotheLiomA Patients: A Single Arm Phase II Study (PEMMELA). *Journal of Thoracic Oncology*, *17*(9), S13-. https://doi.org/10.1016/j.jtho.2022.07.030 |
| Dummer, R., Long, G. V, Robert, C., Tawbi, H. A., Flaherty, K. T., Ascierto, P. A., Nathan, P. D., Rutkowski, P., Leonov, O., Dutriaux, C., Mandalà, M., Lorigan, P., Ferrucci, P. F., Grob, J. J., Meyer, N., Gogas, H., Stroyakovskiy, D., Arance, A., Brase, J. C., … Schadendorf, D. (2022). Randomized Phase III Trial Evaluating Spartalizumab Plus Dabrafenib and Trametinib for BRAF V600–Mutant Unresectable or Metastatic Melanoma. *Journal of Clinical Oncology*, *40*(13), 1428–1438. https://doi.org/10.1200/JCO.21.01601 |
| Dummer, R., Queirolo, P., Abajo Guijarro, A. M., Hu, Y., Wang, D., de Azevedo, S. J., Robert, C., Ascierto, P. A., Chiarion-Sileni, V., Pronzato, P., Spagnolo, F., Mujika Eizmendi, K., Liszkay, G., de la Cruz Merino, L., & Tawbi, H. (2022). Atezolizumab, vemurafenib, and cobimetinib in patients with melanoma with CNS metastases (TRICOTEL): a multicentre, open-label, single-arm, phase 2 study. *The Lancet. Oncology*, *23*(9), 1145–1155. https://doi.org/https://dx.doi.org/10.1016/S1470-2045(22)00452-1 |
| Eng, C., Kim, T. W., Bendell, J., Argilés, G., Tebbutt, N. C., Di Bartolomeo, M., Falcone, A., Fakih, M., Kozloff, M., Segal, N. H., Sobrero, A., Yan, Y., Chang, I., Uyei, A., Roberts, L., Ciardiello, F., Ahn, J. B., Asselah, J., Badarinath, S., … Young, R. (2019). Atezolizumab with or without cobimetinib versus regorafenib in previously treated metastatic colorectal cancer (IMblaze370): a multicentre, open-label, phase 3, randomised, controlled trial. *The Lancet Oncology*, *20*(6), 849–861. https://doi.org/10.1016/S1470-2045(19)30027-0 |
| Fakih, M., Raghav, K. P. S., Chang, D. Z., Bendell, J. C., Larson, T., Cohn, A. L., Huyck, T. K., Cosgrove, D., Fiorillo, J. A., Garbo, L. E., Ravimohan, S., Potter, V., D’Adamo, D., Sharma, N., Wang, Y. A., Coppieters, S., Herpers, M., Soares Viana de Oliveira, C., & Paulson, A. S. (2021). Single-arm, phase 2 study of regorafenib plus nivolumab in patients with mismatch repair-proficient (pMMR)/microsatellite stable (MSS) colorectal cancer (CRC). *Journal of Clinical Oncology*, *39*(15_suppl), 3560. https://doi.org/10.1200/JCO.2021.39.15_suppl.3560 |
| Ferrarotto, R., Bell, D., Feng, L., Li, K., Mott, F., Blumenschein, G. R., De Sousa, L. G., Altan, M., Marques-Piubelli, M. L., Dal Lago, E. A., Kaya, D., Godoy, M., Kupferman, M. E., Glisson, B. S., El-Naggar, A. K., & Elamin, Y. Y. (2022). A phase 2 clinical trial of axitinib and avelumab in patients with recurrent/metastatic adenoid cystic carcinoma (ACC). *Journal of Clinical Oncology*, *40*(16). https://doi.org/10.1200/JCO.2022.40.16_suppl.6019 |
| Ferrucci, P. F., Di Giacomo, A. M., Del Vecchio, M., Atkinson, V., Schmidt, H., Schachter, J., Queirolo, P., Long, G. V, Stephens, R., Svane, I. M., Lotem, M., Abu-Amna, M., Gasal, E., Ghori, R., Diede, S. J., Croydon, E. S., Ribas, A., & Ascierto, P. A. (2020). KEYNOTE-022 part 3: a randomized, double-blind, phase 2 study of pembrolizumab, dabrafenib, and trametinib in &lt;em&gt;BRAF&lt;/em&gt;-mutant melanoma. *Journal for ImmunoTherapy of Cancer*, *8*(2), e001806-. https://doi.org/10.1136/jitc-2020-001806 |
| Galle, P. R., Kim, R. D., Sung, M. W., Harris, W. P., Waldschmidt, D., Cabrera, R., Mueller, U., Nakajima, K., Ishida, T., & El-Khoueiry, A. B. (2020). Updated results of a phase Ib study of regorafenib (REG) plus pembrolizumab (PEMBRO) for first-line treatment of advanced hepatocellular carcinoma (HCC). *Annals of Oncology*, *31*, S691–S692. https://doi.org/10.1016/j.annonc.2020.08.1106 |
| Gao, G., Wang, Y., Ren, S., Liu, Z., Chen, G., Gu, K., Zang, A., Zhao, J., Guo, R., He, J., Lin, X., Pan, Y., Ma, Z., Wang, Z., Fan, M., Liu, Y., Cang, S., Yang, X., Wang, Q., & Zhou, C. (2021). P83.03 Efficacy of Camrelizumab (SHR-1210) Plus Apatinib in Advanced NSCLC with EGFR Mutation. *Journal of Thoracic Oncology*, *16*(3), S654-. https://doi.org/10.1016/j.jtho.2021.01.1198 |
| Gettinger, S., Hellmann, M. D., Chow, L. Q. M., Borghaei, H., Antonia, S., Brahmer, J. R., Goldman, J. W., Gerber, D. E., Juergens, R. A., Shepherd, F. A., Laurie, S. A., Young, T. C., Li, X., Geese, W. J., & Rizvi, N. (2018). Nivolumab Plus Erlotinib in Patients With EGFR-Mutant Advanced NSCLC. *Journal of Thoracic Oncology : Official Publication of the International Association for the Study of Lung Cancer*, *13*(9), 1363–1372. https://doi.org/https://dx.doi.org/10.1016/j.jtho.2018.05.015 |
| Girardi, D. M., Niglio, S. A., Mortazavi, A., Nadal, R., Lara, P., Pal, S. K., Saraiya, B., Cordes, L., Ley, L., Ortiz, O. S., Cadena, J., Diaz, C., Bagheri, H., Redd, B., Steinberg, S. M., Costello, R., Chan, K. S., Lee, M.-J., Lee, S., … Apolo, A. B. (2022). Cabozantinib plus Nivolumab Phase I Expansion Study in Patients with Metastatic Urothelial Carcinoma Refractory to Immune Checkpoint Inhibitor Therapy. *Clinical Cancer Research : An Official Journal of the American Association for Cancer Research*, *28*(7), 1353–1362. https://doi.org/https://dx.doi.org/10.1158/1078-0432.CCR-21-3726 |
| Gomez-Roca, C., Yanez, E., Im, S. A., Alvarez, E. C., Senellart, H., Doherty, M., García-Corbacho, J., Lopez, J. S., Basu, B., Maurice-Dror, C., Gill, S. S., Ghori, R., Kubiak, P., Jin, F., Norwood, K. G., & Chung, H. C. (2021). LEAP-005: A phase II multicohortstudy of lenvatinib pluspembrolizumab in patients withpreviously treated selected solidtumors-Results from thecolorectal cancer cohort. *Journal of Clinical Oncology*, *39*(3 SUPPL). https://doi.org/10.1200/JCO.2021.39.3_suppl.94 |
| González-Martín, A., Chung, H., Saada-Bouzid, E., Yanez, E., Senellart, H., Cassier, P. A., Basu, B., Ghori, R., Kubiak, P., Smith, A., Norwood, K., & Lwin, Z. (2020). Efficacy and safety of lenvatinib plus pembrolizumab in patients with previously treated ovarian cancer in the multicohort phase 2 leap-005 study. *International Journal of Gynecological Cancer*, *30*(SUPPL 3), A1–A2. https://doi.org/10.1136/ijgc-2020-IGCS.2 |
| Gutzmer, R., Stroyakovskiy, D., Gogas, H., Robert, C., Lewis, K., Protsenko, S., Pereira, R. P., Eigentler, T., Rutkowski, P., Demidov, L., Manikhas, G. M., Yan, Y., Huang, K.-C., Uyei, A., McNally, V., McArthur, G. A., & Ascierto, P. A. (2020). Atezolizumab, vemurafenib, and cobimetinib as first-line treatment for unresectable advanced <em>BRAF</em>^V600^ mutation-positive melanoma (IMspire150): primary analysis of the randomised, double-blind, placebo-controlled, phase 3 trial. *The Lancet*, *395*(10240), 1835–1844. https://doi.org/10.1016/S0140-6736(20)30934-X |
| Heudobler, D., Villanueva, L., Lwin, Z., Chung, H. C., Gomez-Roca, C., Muñoz, F. L., Yanez, E., Senellart, H., Doherty, M., Garcia-Corbacho, J., Hendifar, A. E., Maurice-Dror, C., Gill, S., Kim, T. W., Penel, N., Ghori, R., Kubiak, P., Jin, F., Norwood, K., & Graham, D. (2021). Lenvatinib plus pembrolizumab for patients with previously treated biliary tract cancers in the multicohort phase 2 LEAP- 005 study. *Oncology Research and Treatment*, *44*(SUPPL 2), 33–34. https://doi.org/10.1159/000518417 |
| Jiang, M., Zhang, C., Hu, Y., Li, T., Yang, G., Wang, G., Zhu, J., Shao, C., Hou, H., Zhou, N., Liu, K., & Zhang, X. (2022). Anlotinib Combined with Toripalimab as Second-Line Therapy for Advanced, Relapsed Gastric or Gastroesophageal Junction Carcinoma. *The Oncologist*, *27*(11), e856–e869. https://doi.org/10.1093/oncolo/oyac136 |
| Ju, W., Xia, R., Zhu, D., Dou, S., Zhu, G., Dong, M., Wang, L., Sun, Q., Zhao, T., Zhou, Z., Liang, S., Huang, Y., Tang, Y., Wu, S., Xia, J., Chen, S., Bai, Y., Li, J., Zhu, Q., & Zhong, L. (2022). A pilot study of neoadjuvant combination of anti-PD-1 camrelizumab and VEGFR2 inhibitor apatinib for locally advanced resectable oral squamous cell carcinoma. *Nature Communications*, *13*(1), 5378. https://doi.org/10.1038/s41467-022-33080-8 |
| Kao, H. F., & Hong, R. L. (2018). Pembrolizumab and afatinib for recurrent or metastatic head and neck squamous cell carcinoma. *Annals of Oncology*, *29*, viii379-. https://doi.org/10.1093/annonc/mdy287.018 |
| Karam, J. A., Msaouel, P., Matin, S. F., Campbell, M. T., Zurita, A. J., Shah, A. Y., Wistuba, I. I., Haymaker, C. L., Marmonti, E., Duose, D. Y., Parra, E. R., Solis, L. M., Laberiano, C., Lozano, M., Abraham, A., Hallin, M., Olson, P., Der-Torossian, H., Tannir, N. M., & Wood, C. G. (2021). A phase II study of sitravatinib (Sitra) in combination with nivolumab (Nivo) in patients (Pts) undergoing nephrectomy for locally-advanced clear cell renal cell carcinoma (accRCC). *Journal of Clinical Oncology*, *39*(6 SUPPL). https://doi.org/10.1200/JCO.2021.39.6-suppl.312 |
| Kawazoe, A., Fukuoka, S., Nakamura, Y., Kuboki, Y., Wakabayashi, M., Nomura, S., Mikamoto, Y., Shima, H., Fujishiro, N., Higuchi, T., Sato, A., Kuwata, T., & Shitara, K. (2020). Lenvatinib plus pembrolizumab in patients with advanced gastric cancer in the first-line or second-line setting (EPOC1706): an open-label, single-arm, phase 2 trial. *The Lancet. Oncology*, *21*(8), 1057–1065. https://doi.org/https://dx.doi.org/10.1016/S1470-2045(20)30271-0 |
| Kim, D.-W., Gadgeel, S., Gettinger, S. N., Riely, G. J., Oxnard, G. R., Mekhail, T., Schmid, P., Dowlati, A., Heist, R. S., Wozniak, A. J., Singh, J., Cha, E., Spahn, J., & Ou, S.-H. I. (2022). Brief Report: Safety and Antitumor Activity of Alectinib Plus Atezolizumab From a Phase 1b Study in Advanced <em>ALK</em>-Positive NSCLC. *JTO Clinical and Research Reports*, *3*(8). https://doi.org/10.1016/j.jtocrr.2022.100367 |
| Kim, R., Imanirad, I., Strosberg, J., Carballido, E., & Kim, D. (2021). PD-2 Final result of phase IB study of regorafenib and nivolumab in mismatch repair proficient advanced refractory colorectal cancer. *Annals of Oncology*, *32*, S199-. https://doi.org/10.1016/j.annonc.2021.05.020 |
| Kudo, M., Motomura, K., Wada, Y., Inaba, Y., Sakamoto, Y., Kurosaki, M., Umeyama, Y., Kamei, Y., Yoshimitsu, J., Fujii, Y., Aizawa, M., Robbins, P. B., & Furuse, J. (2021). Avelumab in Combination with Axitinib as First-Line Treatment in Patients with Advanced Hepatocellular Carcinoma: Results from the Phase 1b VEGF Liver 100 Trial. *Liver Cancer*, *10*(3), 249–259. https://doi.org/https://dx.doi.org/10.1159/000514420 |
| Lan, C., Shen, J., Wang, Y., Li, J., Liu, Z., He, M., Cao, X., Ling, J., Huang, J., Zheng, M., Zou, G., Yan, H., Liu, Q., Yang, F., Wei, W., Deng, Y., Xiong, Y., & Huang, X. (2020). Camrelizumab Plus Apatinib in Patients With Advanced Cervical Cancer (CLAP): A Multicenter, Open-Label, Single-Arm, Phase II Trial. *Journal of Clinical Oncology : Official Journal of the American Society of Clinical Oncology*, *38*(34), 4095–4106. https://doi.org/https://dx.doi.org/10.1200/JCO.20.01920 |
| Lan, C., Zhao, J., Yang, F., Li, R., Huang, Y., Wang, J., Zhao, W., Zhang, L., Liu, C., Bi, X., Jin, H., Meng, J., & Huang, X. (2021). Anlotinib in combination with TQB2450 in patients with recurrent ovarian cancer (ACTION): A multicenter, single-arm, openlabel, phase Ib trial. *Journal of Clinical Oncology*, *39*(15 SUPPL). https://doi.org/10.1200/JCO.2021.39.15_suppl.5557 |
| Lee, C. H., Voss, M. H., Carlo, M. I., Chen, Y. B., Reznik, E., Knezevic, A., Lefkowitz, R. A., Shapnik, N., Tassone, D., Dadoun, C., Shah, N. J., Owens, C. N., McHugh, D. J., Aggen, D. H., Laccetti, A. L., Kotecha, R., Feldman, D. R., & Motzer, R. J. (2021). Nivolumab plus cabozantinib in patients with non-clear cell renal cell carcinoma: Results of a phase 2 trial. *Journal of Clinical Oncology*, *39*(15 SUPPL). https://doi.org/10.1200/JCO.2021.39.15-suppl.4509 |
| Lee, C.-H., Shah, A. Y., Rasco, D., Rao, A., Taylor, M. H., Di Simone, C., Hsieh, J. J., Pinto, A., Shaffer, D. R., Girones Sarrio, R., Cohn, A. L., Vogelzang, N. J., Bilen, M. A., Gunnestad Ribe, S., Goksel, M., Tennøe, Ø. K., Richards, D., Sweis, R. F., Courtright, J., … Motzer, R. J. (2021). Lenvatinib plus pembrolizumab in patients with either treatment-naive or previously treated metastatic renal cell carcinoma (Study 111/KEYNOTE-146): a phase 1b/2 study. *The Lancet Oncology*, *22*(7), 946–958. https://doi.org/10.1016/S1470-2045(21)00241-2 |
| Lheureux, S., Matei, D. E., Konstantinopoulos, P. A., Wang, B. X., Gadalla, R., Block, M. S., Jewell, A., Gaillard, S. L., McHale, M., McCourt, C., Temkin, S., Girda, E., Backes, F. J., Werner, T. L., Duska, L., Kehoe, S., Colombo, I., Wang, L., Li, X., … Fleming, G. F. (2022). Translational randomized phase II trial of cabozantinib in combination with nivolumab in advanced, recurrent, or metastatic endometrial cancer. *Journal for ImmunoTherapy of Cancer*, *10*(3), e004233-. https://doi.org/10.1136/jitc-2021-004233 |
| Lin, J., Yang, X., Long, J., Zhao, S., Mao, J., Wang, D., Bai, Y., Bian, J., Zhang, L., Yang, X., Wang, A., Xie, F., Shi, W., Yang, H., Pan, J., Hu, K., Guan, M., Zhao, L., Huo, L., … Zhao, H. (2020). Pembrolizumab combined with lenvatinib as non-first-line therapy in patients with refractory biliary tract carcinoma. *Hepatobiliary Surgery and Nutrition*, *9*(4), 414–424. https://hbsn.amegroups.com/article/view/43240 |
| Llovet, J., Finn, R., Ikeda, M., Sung, M., Baron, A., Kudo, M., Okusaka, T., Kobayashi, M., Kumada, H., Kaneko, S., Pracht, M., Mamontov, K., Meyer, T., Mody, K., Kubota, T., Dutcus, C., Saito, K., Siegel, A., Dubrovsky, L., … Zhu, A. X. (2019). A phase 1b trial of lenvatinib (LEN) plus pembrolizumab (PEMBRO) in unresectable hepatocellular carcinoma (uHCC): Updated results. *Asia-Pacific Journal of Clinical Oncology*, *15*(SUPPL 9), 189–190. https://doi.org/10.1111/ajco.13263 |
| Makker, V., Taylor, M. H., Aghajanian, C., Oaknin, A., Mier, J., Cohn, A. L., Romeo, M., Bratos, R., Brose, M. S., DiSimone, C., Messing, M., Stepan, D. E., Dutcus, C. E., Wu, J., Schmidt, E. V, Orlowski, R., Sachdev, P., Shumaker, R., & Casado Herraez, A. (2020). Lenvatinib Plus Pembrolizumab in Patients With Advanced Endometrial Cancer. *Journal of Clinical Oncology : Official Journal of the American Society of Clinical Oncology*, *38*(26), 2981–2992. https://doi.org/https://dx.doi.org/10.1200/JCO.19.02627 |
| Marandino, L., Raggi, D., Calareso, G., Alessi, A., Colecchia, M., Martini, A., Briganti, A., Montorsi, F., Madison, R., Ross, J. S., & Necchi, A. (2021). Cabozantinib Plus Durvalumab in Patients With Advanced Urothelial Carcinoma After Platinum Chemotherapy: Safety and Preliminary Activity of the Open-Label, Single-Arm, Phase 2 ARCADIA Trial. *Clinical Genitourinary Cancer*, *19*(5), 457–465. https://doi.org/10.1016/j.clgc.2021.04.001 |
| Martin-Broto, J., Hindi, N., Grignani, G., Martinez-Trufero, J., Redondo, A., Valverde, C., Stacchiotti, S., Lopez-Pousa, A., D’Ambrosio, L., Gutierrez, A., Perez-Vega, H., Encinas-Tobajas, V., de Alava, E., Collini, P., Pena-Chilet, M., Dopazo, J., Carrasco-Garcia, I., Lopez-Alvarez, M., Moura, D. S., & Lopez-Martin, J. A. (2020). Nivolumab and sunitinib combination in advanced soft tissue sarcomas: a multicenter, single-arm, phase Ib/II trial. *Journal for ImmunoTherapy of Cancer*, *8*(2). https://doi.org/https://dx.doi.org/10.1136/jitc-2020-001561 |
| Meng, X., Wu, T., Hong, Y., Fan, Q., Ren, Z., Guo, Y., Yang, X., Shi, P., Yang, J., Yin, X., Luo, Z., Xia, J., Zhou, Y., Xu, M., Liu, E., Jiang, G., Li, S., Zhao, F., Ma, C., … Wang, F. (2022). Camrelizumab plus apatinib as second-line treatment for advanced oesophageal squamous cell carcinoma (CAP 02): a single-arm, open-label, phase 2 trial. *The Lancet Gastroenterology & Hepatology*, *7*(3), 245–253. https://doi.org/10.1016/S2468-1253(21)00378-2 |
| Motzer, R. J., Penkov, K., Haanen, J. B. A. G., Rini, B. I., Albiges, L., Campbell, M. T., Kollmannsberger, C. K., Negrier, S., Uemura, M., Lee, J. L., Gurney, H., Berger, R., Schmidinger, M., Larkin, J., Atkins, M. B., Wang, J., Robbins, P. B., Chudnovsky, A., Di Pietro, A., & Choueiri, T. K. (2018). JAVELIN renal 101: A randomized, phase III study of avelumab 1 axitinib vs sunitinib as first-line treatment of advanced renal cell carcinoma (aRCC). *Annals of Oncology*, *29*, viii724-. https://doi.org/10.1093/annonc/mdy424.036 |
| Neal, J. W., Lim, F. L., Aix, S. P., Viteri, S., Santoro, A., Spencer, K., Fang, B., Khrizman, P., Kim, J., Subbiah, V., Sudhagoni, R., Samaraweera, L., Andrianova, L., & Felip, E. (2022). EP08.02-081 Cabozantinib Plus Atezolizumab in First or Second-Line Advanced NSCLC and Previously-Treated EGFR Mutant Advanced NSCLC. *Journal of Thoracic Oncology*, *17*(9), S439-. https://doi.org/10.1016/j.jtho.2022.07.763 |
| Oliva, M., Chepeha, D., Araujo, D. V, Diaz-Mejia, J. J., Olson, P., Prawira, A., Spreafico, A., Bratman, S. V, Shek, T., de Almeida, J., R Hansen, A., Hope, A., Goldstein, D., Weinreb, I., Smith, S., Perez-Ordonez, B., Irish, J., Torti, D., Bruce, J. P., … Siu, L. (2021). Antitumor immune effects of preoperative sitravatinib and nivolumab in oral cavity cancer: SNOW window-of-opportunity study. *Journal for ImmunoTherapy of Cancer*, *9*(10). https://doi.org/https://dx.doi.org/10.1136/jitc-2021-003476 |
| Pal, S. K., Agarwal, N., Singh, P., Necchi, A., McGregor, B. A., Hauke, R. J., Powles, T., Suárez, C., Herpen, C. M. L. V, Vaishampayan, U. N., Sudhagoni, R., Curran, D., Andrianova, L., & Loriot, Y. (2022). Cabozantinib (C) in combination with atezolizumab (A) in urothelial carcinoma (UC): Results from Cohorts 3, 4, 5 of the COSMIC-021 study. *Journal of Clinical Oncology*, *40*(16). https://doi.org/10.1200/JCO.2022.40.16_suppl.4504 |
| Plimack, E. R., Rini, B. I., Stus, V., Gafanov, R., Waddell, T., Nosov, D., Pouliot, F., Soulieres, D., Melichar, B., Vynnychenko, I., Azevedo, S. J., Borchiellini, D., McDermott, R. S., Bedke, J., Tamada, S., Yin, L., Chen, M., Molife, L. R., Atkins, M. B., & Powles, T. (2020). Pembrolizumab plus axitinib versus sunitinib as first-line therapy for advanced renal cell carcinoma (RCC): Updated analysis of KEYNOTE-426. *Journal of Clinical Oncology*, *38*(15). https://doi.org/10.1200/JCO.2020.38.15_suppl.5001 |
| Qu, Y.-Y., Zhang, H.-L., Guo, H., Luo, H., Zou, Q., Xing, N., Xia, S., Sun, Z., Zhang, X., He, C., Cai, J., Zhang, X., Wang, Q., & Ye, D.-W. (2021). Camrelizumab plus Famitinib in Patients with Advanced or Metastatic Renal Cell Carcinoma: Data from an Open-label, Multicenter Phase II Basket Study. *Clinical Cancer Research*, *27*(21), 5838–5846. https://doi.org/10.1158/1078-0432.CCR-21-1698 |
| Ren, S., He, J., Fang, Y., Chen, G., Ma, Z., Chen, J., Guo, R., Lin, X., Yao, Y., Wu, G., Wang, Q., & Zhou, C. (2022). Camrelizumab Plus Apatinib in Treatment-Naive Patients With Advanced Nonsquamous NSCLC: A Multicenter, Open-Label, Single-Arm, Phase 2 Trial. *JTO Clinical and Research Reports*, *3*(5). https://doi.org/10.1016/j.jtocrr.2022.100312 |
| Rizvi, N. A., Chow, L. Q. M., Borghaei, H., Shen, Y., Harbison, C., Alaparthy, S., Chen, A. C., & Gettinger, S. N. (2014). Safety and response with nivolumab (anti-PD-1; BMS-936558, ONO-4538) plus erlotinib in patients (pts) with epidermal growth factor receptor mutant (EGFR MT) advanced NSCLC. *Journal of Clinical Oncology*, *32*(15). https://www.embase.com/search/results?subaction=viewrecord&id=L71526285&from=export |
| Spigel, D. R., Reynolds, C., Waterhouse, D., Garon, E. B., Chandler, J., Babu, S., Thurmes, P., Spira, A., Jotte, R., Zhu, J., Lin, W. H., & Blumenschein Jr., G. (2018). Phase 1/2 Study of the Safety and Tolerability of&#xa0;Nivolumab Plus Crizotinib for the First-Line Treatment of Anaplastic Lymphoma Kinase Translocation &#x2014; Positive Advanced Non&#x2013;Small Cell&#xa0;Lung Cancer (CheckMate 370). *Journal of Thoracic Oncology*, *13*(5), 682–688. https://doi.org/10.1016/j.jtho.2018.02.022 |
| Sullivan, R. J., Hamid, O., Gonzalez, R., Infante, J. R., Patel, M. R., Hodi, F. S., Lewis, K. D., Tawbi, H. A., Hernandez, G., Wongchenko, M. J., Chang, Y. M., Roberts, L., Ballinger, M., Yan, Y., Cha, E., & Hwu, P. (2019). Atezolizumab plus cobimetinib and vemurafenib in BRAF-mutated melanoma patients. *Nature Medicine*. https://doi.org/10.1038/s41591-019-0474-7 |
| Sun, Y., Qu, W., Sun, M., Zhou, J., Bi, X., & Zhou, A. (2022). 1743P ALTN-AK105-II-02 cohort 4: A phase II study of penpulimab plus anlotinib in patients (pts) with previously treated locally advanced or metastatic urothelial carcinoma (UC). *Annals of Oncology*, *33*, S1334-. https://doi.org/10.1016/j.annonc.2022.07.1821 |
| Taylor, M. H., Lee, C.-H., Makker, V., Rasco, D., Dutcus, C. E., Wu, J., Stepan, D. E., Shumaker, R. C., & Motzer, R. J. (2020). Phase IB/II Trial of Lenvatinib Plus Pembrolizumab in Patients With Advanced Renal Cell Carcinoma, Endometrial Cancer, and Other Selected Advanced Solid Tumors. *Journal of Clinical Oncology*, *38*(11), 1154–1163. https://doi.org/10.1200/JCO.19.01598 |
| Villanueva, L., Lwin, Z., Chung, H. C., Gomez-Roca, C., Longo, F., Yanez, E., Senellart, H., Doherty, M., García-Corbacho, J., Hendifar, A. E., Maurice-Dror, C., Gill, S. S., Kim, T. W., Heudobler, D., Penel, N., Ghori, R., Kubiak, P., Jin, F., Norwood, K. G., & Graham, D. (2021). Lenvatinib plus pembrolizumabfor patients with previouslytreated biliary tract cancers in themulticohort phase II LEAP-005study. *Journal of Clinical Oncology*, *39*(3 SUPPL). https://doi.org/10.1200/JCO.2021.39.3-suppl.321 |
| Vogel, A., Siegler, G. M., Siebler, J., Lindig, U., Schultheiß, M., Müller, T., Simon, H., Jöckel, C., Mueller, D. W., Al-Batran, S. E., Saborowski, A., & De Toni, E. N. (2022). IMMUNIB trial (AIO-HEP-0218/ass): A single-arm, phase II study evaluating safety and efficacy of immunotherapy nivolumab in combination with lenvatinib in advancedstage hepatocellular carcinoma (HCC). *Journal of Clinical Oncology*, *40*(16). https://doi.org/10.1200/JCO.2022.40.16_suppl.4107 |
| Wang, D., Yang, X., Long, J., Lin, J., Mao, J., Xie, F., Wang, Y., Wang, Y., Xun, Z., Bai, Y., Yang, X., Guan, M., Pan, J., Seery, S., Sang, X., & Zhao, H. (2021). The Efficacy and Safety of Apatinib Plus Camrelizumab in Patients With Previously Treated Advanced Biliary Tract Cancer: A Prospective Clinical Study. *Frontiers in Oncology*, *11*. https://doi.org/10.3389/fonc.2021.646979 |
| Wei, W., Ban, X., Yang, F., Li, J., Cheng, X., Zhang, R., Huang, X., Huang, Y., Li, Q., Qiu, Y., Zheng, M., Zhu, X., & Li, J. (2022). Phase II trial of efficacy, safety and biomarker analysis of sintilimab plus anlotinib for patients with recurrent or advanced endometrial cancer. *Journal for ImmunoTherapy of Cancer*, *10*(5). https://doi.org/https://dx.doi.org/10.1136/jitc-2021-004338 |
| Wilky, B. A., Trucco, M. M., Kolonias, D., Wieder, E., Subhawong, T., Rosenberg, A., Kwon, D., Park, W., Florou, V., Kerr, D. A., Sfakianaki, E., Merchan, J. R., Komanduri, K., & Trent, J. C. (2018). A phase II trial of axitinib plus pembrolizumab for patients with advanced alveolar soft part sarcoma (ASPS) and other soft tissue sarcomas (STS). *Journal of Clinical Oncology*, *36*(15). https://doi.org/10.1200/JCO.2018.36.15_suppl.11547 |
| Xie, L., Xu, J., Sun, X., Guo, W., Gu, J., Liu, K., Zheng, B., Ren, T., Huang, Y., Tang, X., Yan, T., Yang, R., Sun, K., Shen, D., & Li, Y. (2020). Apatinib plus camrelizumab (anti-PD1 therapy, SHR-1210) for advanced osteosarcoma (APFAO) progressing after chemotherapy: a single-arm, open-label, phase 2 trial. *Journal for ImmunoTherapy of Cancer*, *8*(1), e000798-. https://doi.org/10.1136/jitc-2020-000798 |
| Xu, Q., Wang, J., Sun, Y., Lin, Y., Liu, J., Zhuo, Y., Huang, Z., Huang, S., Chen, Y., Chen, L., Ke, M., Li, L., Li, Z., Pan, J., Song, Y., Liu, R., & Chen, C. (2022). Efficacy and Safety of Sintilimab Plus Anlotinib for PD-L1–Positive Recurrent or Metastatic Cervical Cancer: A Multicenter, Single-Arm, Prospective Phase II Trial. *Journal of Clinical Oncology*, *40*(16), 1795–1805. https://doi.org/10.1200/JCO.21.02091 |
| Yang, J. C. H., Luft, A., De La Mora Jiménez, E., Lee, J. S., Koralewski, P., Karadurmus, N., Sugawara, S., Livi, L., Basappa, N. S., Quantin, X., Dudnik, J., Moran Ortiz, D., Mekhail, T., Okpara, C. E., Zimmer, Z., Samkari, A., Bhagwati, N., & Csőszi, T. (2021). Pembrolizumab (Pembro) with or without lenvatinib (Lenva) in first-line metastatic NSCLC with PD-L1 TPS ≥1% (LEAP-007): A phase III, randomized, double-blind study. *Annals of Oncology*, *32*, S1429–S1430. https://doi.org/10.1016/j.annonc.2021.10.139 |

**Supplementary Table 1 – Included Studies**

| **Disease Subgroup** | **ICI Target** | **TKI Target** |
| --- | --- | --- |
| **Variable** | **Variable** | **Variable** |
| 1 GI | PD-1 | Multitargeted TKI |
| 2 Skin | PD-L1* | VEGF Specific |
| 3 Breast |  | Oncogenic driver target** |
| 4 Head/Neck + Thyroid |  |  |
| 5 Thoracic |  |  |
| 6 Gynae |  |  |
| 7 GU |  |  |
| 8 Brain |  |  |
| 9 Sarcoma |  |  |

**Supplementary Table 2 – Covariates & Variables used for meta-regression**

*****In one study, patients received a CTLA-4 & PD-1 therapy – this was excluded from the analysis

**Oncogenic driver = ALK, EGFR, BRAF, MEK (Grouped together to facilitate meta-regression)

| **Title** | **Citation** | **Bias** |
| --- | --- | --- |
| Axitinib in combination with pembrolizumab in patients with advanced renal cell cancer: a non-randomised, open-label, dose-finding, and dose-expansion phase 1b trial | Atkins et al, 2018 | 35 |
| Atezolizumab with or without cobimetinib versus regorafenib in previously treated metastatic colorectal cancer (IMblaze370): a multicentre, open-label, phase 3, randomised, controlled trial | Eng C et al, 2018 | 35 |
| Safety and response with nivolumab (anti-PD-1; BMS-936558, ONO-4538) plus erlotinib in patients (pts) with epidermal growth factor receptor mutant (EGFR MT) advanced NSCLC | Gettinger et al, 2018 | 34 |
| JAVELIN renal 101: A randomized, phase III study of avelumab 1 axitinib vs sunitinib as first-line treatment of advanced renal cell carcinoma (aRCC) | Motzer et al, 2019 | 33 |
| Efficacy and safety of lenvatinib plus pembrolizumab in patients with previously treated ovarian cancer in the multicohort phase 2 leap-005 study | Gonzalez Martin et al, 2020 | 14 |
| Axitinib plus avelumab in the treatment of recurrent glioblastoma: a stratified, open-label, single-center phase 2 clinical trial (GliAvAx) | Awada G et al, 2020 | 34 |
| Lenvatinib Plus Pembrolizumab in Patients With Advanced Endometrial Cance | Makker et al, 2020 | 36 |
| KEYNOTE-022 part 3: a randomized, double-blind, phase 2 study of pembrolizumab, dabrafenib, and trametinib in &l BRAF & mutant melanoma | Ferruci et al, 2020 | 33 |
| Atezolizumab, vemurafenib, and cobimetinib as first-line treatment for unresectable advanced BRAFV600 mutation positive melanoma (IMspire150): primary analysis of the randomised, double-blind, placebo-controlled, phase 3 trial | Gutzmer et al, 2020 | 35 |
| Lenvatinib plus pembrolizumab in patients with advanced gastric cancer in the first-line or second-line setting (EPOC1706): an open-label, single-arm, phase 2 trial | Kawazoe A et al, 2020 | 37 |
| Camrelizumab Plus Apatinib in Patients With Advanced Cervical Cancer (CLAP): A Multicenter, Open-Label, Single-Arm, Phase II Trial | Lan A et al, 2020 | 36 |
| Pembrolizumab combined with lenvatinib as non-first-line therapy in patients with refractory biliary tract carcinoma | Lin M et al, 2020 | 32 |
| A phase 1b trial of lenvatinib (LEN) plus pembrolizumab (PEMBRO) in unresectable hepatocellular carcinoma (uHCC): Updated results | Finn RS et al, 2020 | 37 |
| Lenvatinib Plus Pembrolizumab in Patients With Advanced Endometrial Cancer | Makker et al, 2020 | 33 |
| Nivolumab and sunitinib combination in advanced soft tissue sarcomas: a multicenter, single-arm, phase Ib/II trial | Martin Broto et al, 2020 | 36 |
| Pembrolizumab plus Axitinib versus Sunitinib for Advanced Renal-Cell Carcinoma | Rini et al, 2020 | 36 |
| Camrelizumab Plus Apatinib in Treatment-Naive Patients With Advanced Nonsquamous NSCLC: A Multicenter, Open-Label, Single-Arm, Phase 2 Trial | Ren et al, 2020 | 33 |
| Phase 1/2 Study of the Safety and Tolerability of Nivolumab Plus Crizotinib for the First-Line Treatment of Anaplastic Lymphoma Kinase Translocation - Positive Advanced Non-Small Cell Lung Cancer (CheckMate 370) | Spigel et al, 2020 | 33 |
| Atezolizumab plus cobimetinib and vemurafenib in BRAF-mutated melanoma patients | Sullivan et al, 2020 | 35 |
| Atezolizumab plus cobimetinib and vemurafenib in BRAF-mutated melanoma patients | Sullivan et al, 2020 | 35 |
| Phase IB/II Trial of Lenvatinib Plus Pembrolizumab in Patients With Advanced Renal Cell Carcinoma, Endometrial Cancer, and Other Selected Advanced Solid Tumors | Taylor et al, 2020 | 36 |
| The Efficacy and Safety of Apatinib Plus Camrelizumab in Patients With Previously Treated Advanced Biliary Tract Cancer: A Prospective Clinical Study | Zhao et al, 2020 | 32 |
| Apatinib plus camrelizumab (anti-PD1 therapy, SHR-1210) for advanced osteosarcoma (APFAO) progressing after chemotherapy: a single-arm, open-label, phase 2 trial | Xie et al, 2020 | 34 |
| Phase II Study of TRIplet combination Nivolumab (N) with Dabrafenib (D) and Trametinib (T) (TRIDeNT) in patients (pts) with PD-1 naïve or refractory BRAF-mutated metastatic melanoma (MM) with or without active brain metastases | Burton et al, 2021 | 16 |
| LEAP-005: A phase II multicohort study of lenvatinib plus pembrolizumab in patients with previously treated selected solid tumors-Results from the gastric cancer cohort | Chung et al, 2021 | 15 |
| Lenvatinib plus pembrolizumab for previously treated, advanced triple-negative breastcancer: Early results from the multicohort phase 2 LEAP-005 study | Chung et al, 2021 | 16 |
| Single-arm, phase 2 study of regorafenib plus nivolumab in patients with mismatch repair-proficient (pMMR)/microsatellite stable (MSS) colorectal cancer (CRC) | Faikh et al, 2021 | 16 |
| Updated results of a phase Ib study of regorafenib (REG) plus pembrolizumab (PEMBRO) for first-line treatment of advanced hepatocellular carcinoma (HCC) | Galle P et al, 2021 | 14 |
| LEAP-005: A phase II multicohort study of lenvatinib plus pembrolizumab in patients with previously treated selected solid tumors-Results from thecolorectal cancer cohort | Gomez Roca et al, 2021 | 15 |
| Lenvatinib plus pembrolizumab for patients with previously treated biliary tract cancers in the multicohort phase 2 LEAP- 005 study | Heudobler et al, 2021 | 14 |
| A phase II study of sitravatinib (Sitra) in combination with nivolumab (Nivo) in patients (Pts) undergoing nephrectomy for locally-advanced clear cell renal cell carcinoma (accRCC) | Karam et al, 2021 | 15 |
| Pembrolizumab (Pembro) with or without lenvatinib (Lenva) in first-line metastatic NSCLC with PD-L1 TPS ≥1% (LEAP-007): A phase III, randomized, double-blind study | Yang et al, 2021 | 15 |
| Nivolumab (NIVO) + ipilimumab (IPI) + cabozantinib (CABO) combination therapy in patients (pts) with advanced hepatocellular carcinoma (aHCC): Results from CheckMate 040 | Yau et al, 2021 | 15 |
| Nivolumab in combination with cabozantinib for metastatic triple-negative breast cancer: a phase II and biomarker study | Barrousa-Sousa R et al, 2021 | 34 |
| Sitravatinib and nivolumab for resectable Oral cavity squamous cell carcinoma window of opportunity study (SNOW) | Snow et al, 2021 | 37 |
| Lenvatinib plus Pembrolizumab or Everolimus for Advanced Renal Cell Carcinoma | Choueiri T et al, 2021 | 33 |
| Phase 1b Study of Sintilimab Plus Anlotinib as First-line Therapy in Patients With Advanced NSCLC | Chu T et al, 2021 | 35 |
| Avelumab in Combination with Axitinib as First-Line Treatment in Patients with Advanced Hepatocellular Carcinoma: Results from the Phase 1b VEGF Liver 100 Trial | Kudo L et al, 2021 | 36 |
| Lenvatinib plus pembrolizumab in patients with either treatment-naive or previously treated metastatic renal cell carcinoma (Study 111/KEYNOTE-146): a phase 1b/2 study | Lee CH et al, 2021 | 35 |
| Cabozantinib Plus Durvalumab in Patients With Advanced Urothelial Carcinoma After Platinum Chemotherapy: Safety and Preliminary Activity of the Open-Label, Single-Arm, Phase 2 ARCADIA Trial | Marandino et al, 2021 | 35 |
| Camrelizumab plus Famitinib in Patients with Advanced or Metastatic Renal Cell Carcinoma: Data from an Open-label, Multicenter Phase II Basket Study | Rizvi et al, 2021 | 36 |
| A phase 1b multitumor cohort study of cabozantinib plus atezolizumab in advanced solid tumors (COSMIC-021): Results of the colorectal cancer cohort | Abrams et al, 2022 | 9 |
| Lenvatinib/pembrolizumab in metastasized anaplastic thyroid carcinoma (atc): Interim results of the atlep trial | Dierks et al, 2022 | 12 |
| OA04.06 PEMbrolizumab Plus Lenvatinib In Second And Third Line Malignant Pleural MEsotheLiomA Patients: A Single Arm Phase II Study (PEMMELA) | Douma et al, 2022 | 15 |
| A phase 2 clinical trial of axitinib and avelumab in patients with recurrent/metastatic adenoid cystic carcinoma (ACC) | Ferroarotta et al, 2022 | 16 |
| EP08.02-081 Cabozantinib Plus Atezolizumab in First or Second-Line Advanced NSCLC and Previously-Treated EGFR Mutant Advanced NSCLC | Neal et al, 2022 | 16 |
| Cabozantinib (C) in combination with atezolizumab (A) in urothelial carcinoma (UC): Results from Cohorts 3, 4, 5 of the COSMIC-021 study | Pal et al, 2022 | 14 |
| 1743P ALTN-AK105-II-02 cohort 4: A phase II study of penpulimab plus anlotinib in patients (pts) with previously treated locally advanced or metastatic urothelial carcinoma (UC) | Sun et al, 2022 | 14 |
| IMMUNIB trial (AIO-HEP-0218/ass): A single-arm, phase II study evaluating safety and efficacy of immunotherapy nivolumab in combination with lenvatinib in advanced stage hepatocellular carcinoma (HCC) | Vogel et al, 2022 | 19 |
| A phase I/II study of nivolumab and axitinib in patients with advanced renal cell carcinoma | Zibelmann et al, 2022 | 16 |
| Cabozantinib in combination with atezolizumab in patients with metastatic castration-resistant prostate cancer: results from an expansion cohort of a multicentre, open-label, phase 1b trial (COSMIC-021) | Agarwal N et al, 2022 | 31 |
| Phase II LEAP-004 Study of Lenvatinib Plus Pembrolizumab for Melanoma With Confirmed Progression on a Programmed Cell Death Protein-1 or Programmed Death Ligand 1 Inhibitor Given as Monotherapy or in Combination | Arance et al, 2022 | 35 |
| Safety and Efficacy of Sintilimab and Anlotinib as First Line Treatment for Advanced Hepatocellular Carcinoma (KEEP-G04): A Single-Arm Phase 2 Study | Chen X et al, 2022 | 35 |
| Nivolumab + cabozantinib vs sunitinib in first-line treatment for advanced renal cell carcinoma: First results from the randomized phase III CheckMate 9ER trial | Choueiri T et al, 2022 | 34 |
| Avelumab plus axitinib in unresectable or metastatic type B3 thymomas and thymic carcinomas (CAVEATT): a single-arm, multicentre, phase 2 trial | Conforti F et al, 2022 | 36 |
| Phase I expansion study of cabozantinib plus nivolumab (CaboNivo) in metastatic urothelial carcinoma (mUC) patients (pts) with progressive disease following immune checkpoint inhibitor (ICI) therapy | Girardi et al, 2022 | 37 |
| Randomized Phase III Trial Evaluating Spartalizumab Plus Dabrafenib and Trametinib for BRAF V600–Mutant Unresectable or Metastatic Melanoma | Dummer R et al, 2022 | 36 |
| Atezolizumab, vemurafenib, and cobimetinib in patients with melanoma with CNS metastases (TRICOTEL): a multicentre, open-label, single-arm, phase 2 study | Dummer R et al, 2022 | 36 |
| Efficacy and safety of camrelizumab plus apatinib in previously treated patients with advanced non-small cell lung cancer harboring EGFR or ALK genetic aberration | Gao G et al, 2022 | 31 |
| Anlotinib Combined with Toripalimab as Second-Line Therapy for Advanced, Relapsed Gastric or Gastroesophageal Junction Carcinoma | Jiang et al, 2022 | 36 |
| A pilot study of neoadjuvant combination of anti-PD-1 camrelizumab and VEGFR2 inhibitor apatinib for locally advanced resectable oral squamous cell carcinoma | Wu-Tong J, 2022 | 33 |
| Pembrolizumab and afatinib for recurrent or metastatic head and neck squamous cell carcinoma | HF Kao et al, 2022 | 36 |
| Brief Report: Safety and Antitumor Activity of Alectinib Plus Atezolizumab From a Phase 1b Study in Advanced ALK Positive NSCLC | Dong N et al, 2022 | 32 |
| PD-2 Final result of phase IB study of regorafenib and nivolumab in mismatch repair proficient advanced refractory colorectal cancer | Kim R et al, 2022 | 30 |
| Anlotinib in combination with TQB2450 in patients with recurrent ovarian cancer (ACTION): A multicenter, single-arm, openlabel, phase Ib trial | Lan C et al, 2022 | 33 |
| Nivolumab plus cabozantinib in patients with non-clear cell renal cell carcinoma: Results of a phase 2 trial | Lee CH et al, 2022 | 36 |
| Translational randomized phase II trial of cabozantinib in combination with nivolumab in advanced, recurrent, or metastatic endometrial cancer | Lheureux U et al, 2022 | 34 |
| Camrelizumab plus apatinib as second-line treatment for advanced oesophageal squamous cell carcinoma (CAP 02): a single-arm, open-label, phase 2 trial | Meng et al, 2022 | 18 |
| Phase II trial of efficacy, safety and biomarker analysis of sintilimab plus anlotinib for patients with recurrent or advanced endometrial cancer | Wei et al, 2022 | 37 |
| A phase II trial of axitinib plus pembrolizumab for patients with advanced alveolar soft part sarcoma (ASPS) and other soft tissue sarcomas (STS) | Wilky et al, 2022 | 34 |
| Efficacy and Safety of Sintilimab Plus Anlotinib for PD-L1–Positive Recurrent or Metastatic Cervical Cancer: A Multicenter, Single-Arm, Prospective Phase II Trial | Xu et al, 2022 | 35 |
| Phase Ib study of anlotinib combined with TQB2450 in pretreated advanced biliary tract cancer and biomarker analysis | Zhou et al, 2022 | 33 |

**Supplementary Table 2 – Crowe Critical Appraisal Tool Score for each Study**

|  | **Non-small cell lung cancer** | **Hepatobiliary** | **Renal cell carcinoma** |
| --- | --- | --- | --- |
| **Total number of studies** | 8 | 10 | 10 |
| **Phase of studies** |  | - | - |
| Phase 1b | 5 (62.5%) | 4 (40%) | 0 |
| Phase 2 | 2 (25%) | 5 (50%) | 0 |
| Phase 3 | 1 (12.5%) | 1 (10%) | 10 (100%) |
| G3-5 toxicity | 57% (95% CI = 43-69%) | 57% (95% CI = 47 -66%) | 58% (95% CI = 40% -73%) |
| **Therapeutic combinations** |  | - | - |
| Axitinib/Pembrolizumab | - | - | 2 (20%) |
| Axitinib/Avelumab | - | 1 (10%) | 1 (10%) |
| Sitravatinimab/Nivolumab | - |  | 1 (10%) |
| Lenvatinib/Pembrolizumab | 1 (12.5%) | 3 (30%) | 2 (20%) |
| Camrelizumab/Famitinib | - | - | 1 (10%) |
| Axitinib/Nivolumab | - | - | 1 (10%) |
| Cabozantinib/Nivolumab | - | - | 2 (20%) |
| Erlotinib/Nivolumab | 1 (12.5%) | - | - |
| Apatinib/Camrelizumab | 2 (25%) | 1 (10%) | - |
| Crizotinib/Nivolumab | 1 (12.5%) | - | - |
| Anlotinib/Sintilimab | 1 (12.5%) | 1(10%) | - |
| Cabozantinib/Atezolizumab | 1 (12.5%) | - | - |
| Alextinib/Atezoluzumab | 1 (12.5%) | - | - |
| Regorafenib/Pembrolizumab | - | 1 (10%) | - |
| Cabozantinib/Nivolumab/Ipilimumab | - | 1 (10%) | - |
| Lenvatinib/Nivolumab | - | 1 (10%) | - |
| Anlotinib/TQB2450 | - | 1 (10%) | - |

**Supplementary Table 3**

**Search Strategy**

The search strategy utilised the following search terms; Immune checkpoint inhibitors OR immune checkpoint inhibitor, Tyrosine kinase inhibitor OR protein-tyrosine kinases and Neoplasms OR carcinoma OR cancer. In addition to these terms, we also used the MESH terms; Humanized / adverse effects Antineoplastic Agents / therapeutic use Antineoplastic Combined Chemotherapy Protocols / adverse effects. Citations from published work were imported and de-duplicated using Endnote. Forward and backward citation chasing was completed to minimise the possibility of missing relevant studies. MEDLINE, EMBASE, Cochrane Database of Systematic Review and Central Registry of Clinical Trials were searched for publications from 16/8/2002 to 16/8/2022. Conference proceedings (abstracts) were considered eligible and included in our search.
